# Spasmolytic Activity of Plant-Mediated Silver Nanoparticles from Fenugreek Seeds: Impact of Geographic Origin and Extraction Solvent

**DOI:** 10.3390/nano16161011

**Published:** 2026-08-17

**Authors:** Alexandra Ivanova, Simona Ivanova, Dimitar Petrov, Vera Gledacheva, Iliyana Stefanova, Valeri Slavchev, Velichka Yanakieva, Slava Tsoneva, Emiliya Cherneva, Denitsa Momekova, Petia Konsulova, Daniela Karashanova, Stoyanka Nikolova

**Affiliations:** 1 Department of Organic Chemistry, Faculty of Chemistry, University of Plovdiv, 4000 Plovdiv, Bulgaria; ivanova.aleksandra@uni-plovdiv.bg (A.I.); simonaivanova1108@gmail.com (S.I.); 2 Department of Physical Chemistry, Faculty of Chemistry, University of Plovdiv, 4000 Plovdiv, Bulgaria; petrov_d@uni-plovdiv.bg; 3 Department of Medical Physics and Biophysics, Faculty of Pharmacy, Medical University of Plovdiv, 4002 Plovdiv, Bulgaria; iliyana.stefanova@mu-plovdiv.bg (I.S.); valeri.slavchev@mu-plovdiv.bg (V.S.); 4 Department of Microbiology, Technological Faculty, University of Food Technologies, 4002 Plovdiv, Bulgaria; yanakieva_vili@abv.bg; 5 Department of Analytical Chemistry and Computer Chemistry, University of Plovdiv, 4000 Plovdiv, Bulgaria; slava.tsoneva@uni-plovdiv.bg; 6 Department of Chemistry, Faculty of Pharmacy, Medical University of Sofia, 2 Dunav Str., 1000 Sofia, Bulgaria; cherneva@pharmfac.mu-sofia.bg; 7 Department of Pharmaceutical Technology and Biopharmaceutics, Faculty of Pharmacy, Medical University of Sofia, 2 Dunav Street, 1000 Sofia, Bulgaria; dmomekova@pharmfac.mu-sofia.bg; 8 Department of Endocrynology, Medical University of Plovdiv, 4002 Plovdiv, Bulgaria; pkonsulova@gmail.com; 9 Institute of Optical Materials and Technologies “Acad. J. Malinowski”, Bulgarian Academy of Sciences, 1113 Sofia, Bulgaria; dkarashanova@yahoo.com

**Keywords:** fenugreek seeds, geographic origin, solvent, spasmolytic, antimicrobial, antioxidant activity

## Abstract

Background: Fenugreek is a widely used aromatic herb valued as a food ingredient. Fenugreek seeds and leaves are rich in antioxidants and bioactive compounds that support overall health, particularly gastrointestinal function. The present paper describes a green synthesis of silver nanoparticles (AgNPs) utilizing aqueous and ethanolic extracts of fenugreek seeds (*Trigonella foenum-graecum* L.) grown in Bulgaria, Egypt, and India in order to find differences in AgNPs formation depending on geographic region and solvent used for the extraction and to find any changes in their spasmolytic activity. Methods: FT-IR, High-Resolution Transmission Electron Microscopy (HRTEM), DLS, and zeta-potential were used to characterize the produced AgNPs, aiming to verify their stability, size distribution, and formation. The biological activities of these extracts are then compared. Results: The ethanolic extracts were more effective for producing smaller, more homogeneous, and relatively stable AgNPs compared to aqueous extracts. Differences in phytochemical composition, which have a direct impact on the nucleation, development, and stability processes of NPs, could be the reason for the observed variances between plant extracts. AgNPs (aqueous), originated from Bulgaria, exhibited the strongest spasmogenic activity under the applied experimental conditions. The biosynthesized AgNPs also showed a selective antifungal activity against *Aspergillus* and *Penicillium* spp., with modest antibacterial effects. Conclusions: From a biological perspective, all samples’ fenugreek extracts had a minimal impact on gastrointestinal contractility, but the AgNPs derived from these extracts had a greater spasmogenic effect. These findings point to a combination that enhances both functional efficacy and bioavailability. The Bulgarian fenugreek aqueous extract produced AgNPs with the strongest spasmogenic activity of all the preparations examined. The results showed the potential of AgNPs from fenugreek seeds for biomedical applications.

## 1. Introduction

**Figure 1:**
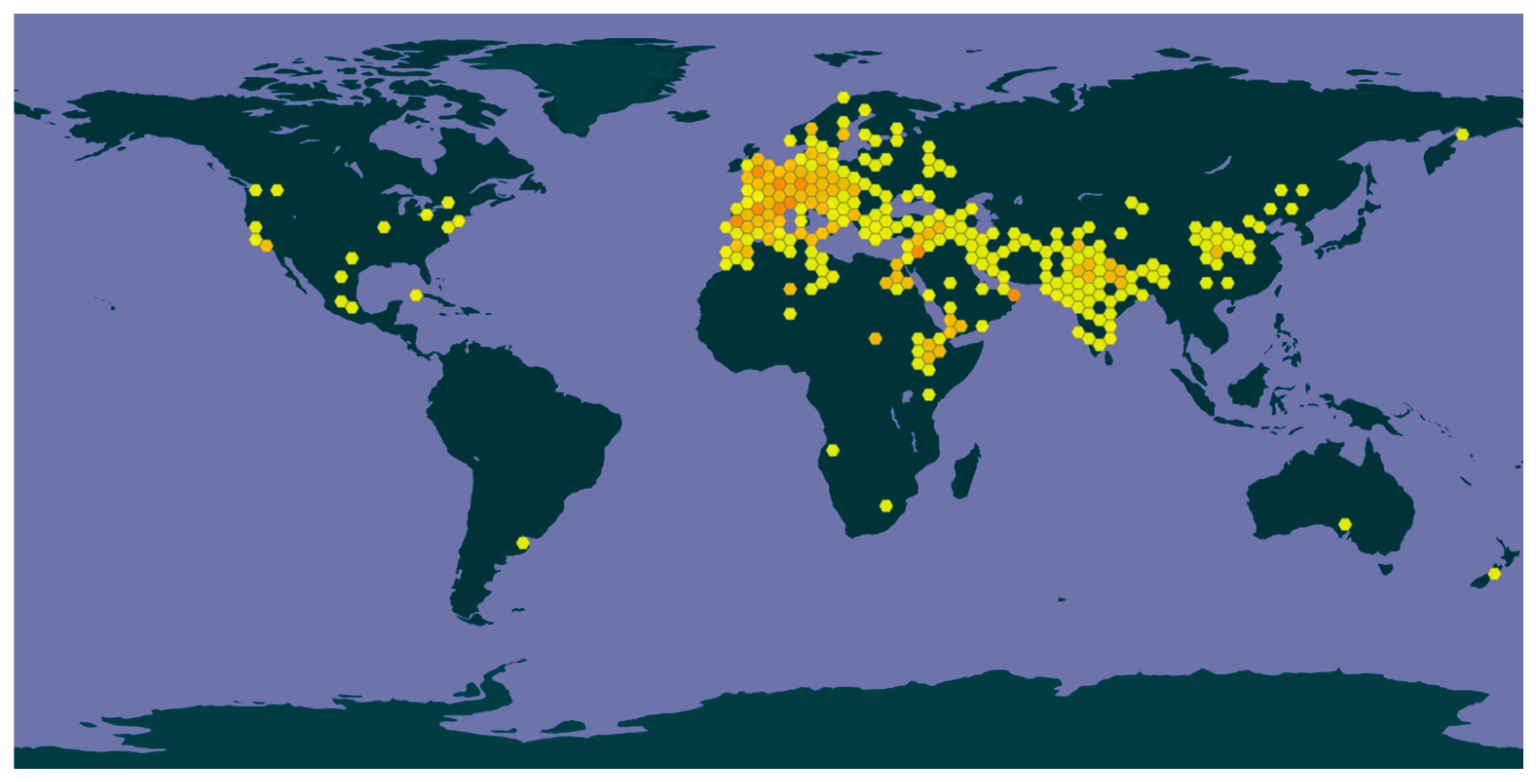
Worldwide distribution of *Trigonella foenum-graecum* [3].

*Trigonella foenum-graecum*, or fenugreek, is popular and widely grown. Although it originated in South-Eastern Europe, Northern Africa, Central Asia, Western Asia, and the Mediterranean region, it is now grown practically everywhere in the world, including India, Canada, Russia, Pakistan, Iran, Afghanistan, Egypt, Morocco, South Eastern Europe, Ethiopia, Chile, Argentina, China, Australia, and the United States (Figure 1) [1].

Despite being the world’s greatest fenugreek producer, India does not make a substantial contribution to the global fenugreek trade due to its high domestic consumption [2].

An annual herbaceous plant, fenugreek usually reaches a height of 50 cm. It features a single, smooth or somewhat hairy stem that is frequently slightly bent. Three tiny, oval to oblong, serrated leaflets that emerge from a single point make up the trifoliate leaves. Its tiny (0.8–1.8 cm) flowers range in color from pale yellow to whitish-purple. The plant yields thin, curving pods that are 3–11 cm long and contain 5–20 tiny, angular seeds (4–6 mm) that range in color from yellowish to brown and have a fragrant, bitter taste [4].

Regional soil, climate, and harvesting conditions affect fenugreek’s nutritional composition [5].

Numerous valuable phytochemicals, including steroidal saponins (diosgenin), fenugreekine (alkaloid), galactomannan (carbohydrate), and 4-hydroxy isoleucine (amino acid), are abundant in fenugreek seed and leaves [6]. More precisely, fenugreek seed contains carbohydrates (45–60%) as in mucilaginous fiber (galactomannans), proteins (20–30%) enriched in tryptophan and lysine, lipids (5–10%) or fixed oil, alkaloids of pyridine type (0.2–0.38%), such as trigonelline, carpaine and gentianine, flavonoids (apigenin, luteolin, quercetin, vitexin, and isovitexin), saponins (0.6–1.7%), and glycosides like yamogenin, tigogenin, and neotigogenin. Alkaloids, steroidal saponins, and oils are responsible for the seeds’ distinctive bitterness.

The presence of these alkaloids, proteins, and carbohydrates makes fenugreek an important medicinal and therapeutic herb. The use of fenugreek in traditional Tibetan, Chinese, Indian Ayurvedic, and Islamic medicine has been documented in ancient books [7]. The effectiveness of this medicinal herb in treating a number of human and animal illnesses has also been conclusively demonstrated by contemporary clinical investigations [8,9]. Fenugreek seed and leaves have been shown in recent studies to be effective in treating a variety of illnesses, including lowering blood sugar and cholesterol levels [6]. The interesting point about fenugreek is the broad range of its therapeutic effects, including pain relief, antidiabetic, antiatherosclerosis, anti-inflammation, carminative, laxative, antispasmodic, anticancer, sexual-desire increasing, astringent, heart tonic, laxative, hypertension decreasing, triglyceride lowering, increasing the quantity of breast milk, and oxytocic properties are reported for this plant [10,11,12].

**Figure 2:**
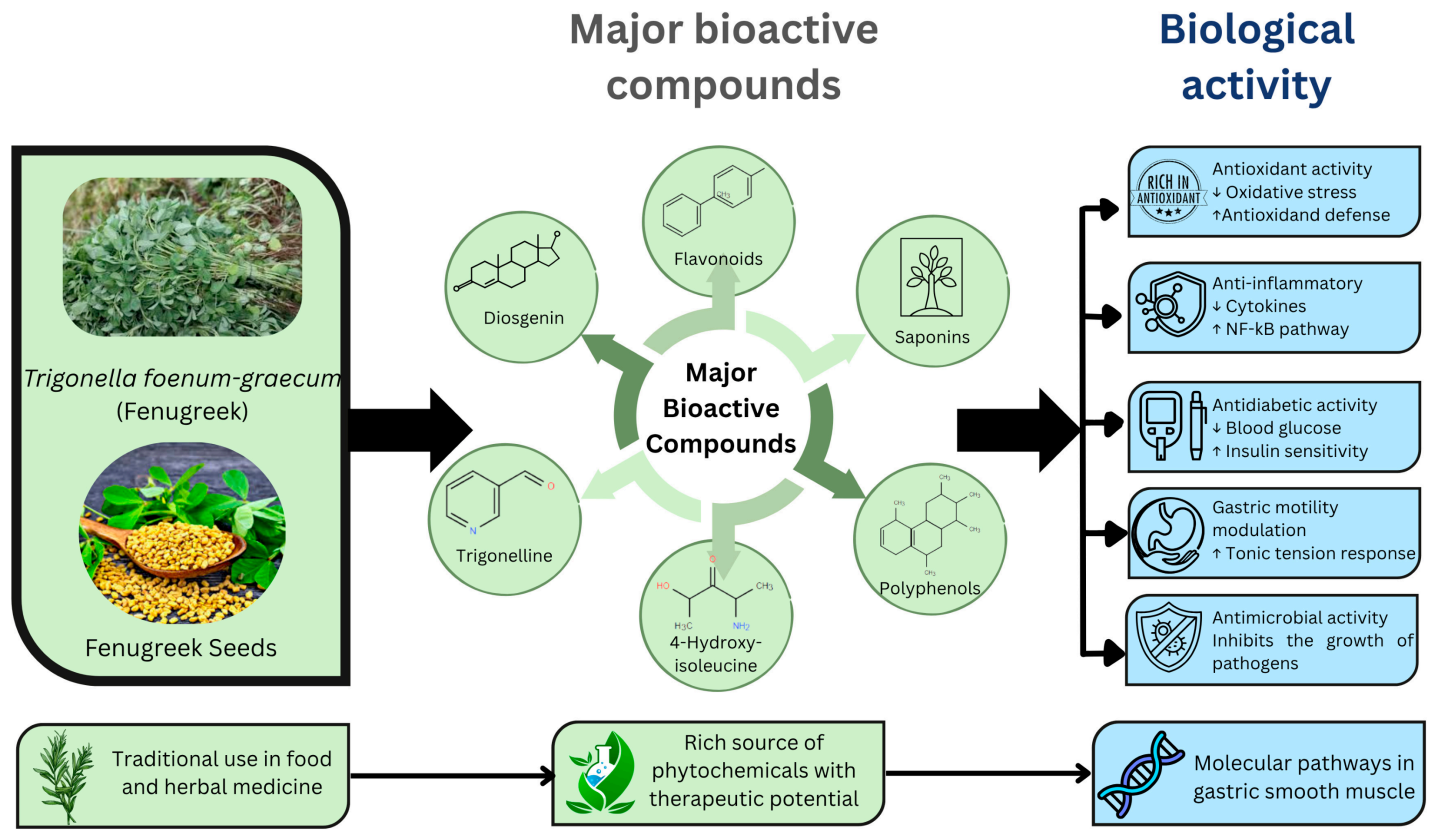
Bioactive compounds of *Trigonella foenum-graecum* and their biological activities: implications for gastric smooth muscles. This image was created with Canva (web-based version), Canva, Australia (https://www.canva.com/bg_bg/), accessed on 15 June 2026.

The principal bioactive compounds, biological activities, and mechanisms potentially involved in the spasmolytic effects of fenugreek are summarized in Figure 2.

Numerous studies have demonstrated fenugreek’s protective effect against gastrointestinal disorders. It has anti-inflammatory, anti-ulcer, gastroprotective, antioxidant, and anti-secretory qualities [13].

The increasing applications of noble metal nanoparticles (NPs) in medical and pharmacological fields, including drug transport, photothermal therapy, radiation, and imaging, have garnered significant attention in recent decades [14]. Among them, silver nanoparticles (AgNPs) have been generated in the largest amounts due to their remarkable biological activity and unique physicochemical characteristics [15,16]. To the best of our knowledge, methods for the environmentally friendly production of AgNPs from fenugreek leaf or seed extract have been documented in the literature; however, their spasmolytic activity has not been evaluated.

In our previous research, we examined the spasmolytic activity of green-synthesized AgNPs from *C. vulgaris* and *Spirulina platensis* extracts [17,18]. A logical extension of this research is the use of fenugreek and its seed extracts for AgNPs synthesis, aiming to compare their spasmolytic activity with the extracts.

The biological characteristics of extracts are known to be greatly impacted by variations in growing conditions, soil composition, and climate [19,20]. Therefore, our main goal was to synthesize AgNPs from fenugreek seeds and to investigate their spasmolytic activity. Additionally, we aimed to determine how the geographic origin of the fenugreek affected the resulting NPs.

## 2. Materials and Methods

All solvents and reagents were purchased from Merck (Merck KGaA, Darmstadt, Germany). Ultrapure water (UPW) was used throughout the experiments (PURELAB Chorus 2+ (ELGA Veolia, High Wycombe, UK) water purification system).

### 2.1. Plant Material

Four samples of *Trigonella foenum-graecum* were analyzed. Two of the samples originated from Egypt, labeled as FutuNatura (EG-1) and MyChef (EG-2); the third sample (BioBock), originated from India (IN-1); and the last one originated from Bulgaria (BG-1), which is cultivated and grown in the Sinitovo village in Bulgaria. All the samples were bought from a local market in Bulgaria and were provided as commercially packaged seeds, according to the manufacturer’s specification. The dried seeds were ground with a mill and then their plant extracts were obtained. Finally, AgNPs were obtained from each batch.

### 2.2. Extract Preparation

A total of 1 g of fenugreek seeds (*Trigonella foenum-graecum*) from each batch was ground, submerged in 10 mL of water or 80% ethanol, and agitated at 40 °C for 50 min. A hydromodule of 1:10 was chosen for the optimal extraction of the active ingredients. The resultant infusion was filtered using Whatman paper.

### 2.3. AgNPs Preparation

Then, 1 mL of extract was mixed with 9 mL of a 10 mM AgNO_3_ solution. The mixture underwent stirring for 4 min at ambient temperature, resulting in a distinct color transition from pale yellow to dark brown.

The theoretical concentration of AgNPs in the colloidal synthesis mixture was calculated using a mathematical method [21,22,23]. Firstly, the average number of atoms per nanoparticle (N) was determined through Equation (1):

(1) $$N=\frac{\left(\pi \rho D^{3}N_{A}\right)}{6M}$$

where π = 3.14, *ρ* = 10.5 g/cm^3^ (density of face centered cubic silver), D = 5.5 × 10^−7^ cm (average diameter of nanoparticles in this study), M = 107.868 g/mol (mass of silver), and N_A_ = 6.023 × 10^23^ (Avogadro’s number).

Secondly, the molar concentration of nanoparticles in the resulting colloidal mixture (C) could be calculated only by considering that all silver ions (Ag^+^) were entirely converted to AgNPs in the biosynthesis process. Equation (2) was applied for the determination of C:

(2) $$C=\frac{N_{T}}{\left(NVN_{A}\right)}$$

where N_T_ = total amount of Ag atoms added as AgNO_3_ (10.0 mM = 0.01 M), N = average number of atoms per nanoparticle, V = reaction volume (0.01 L) and N_A_ = Avogadro’s number [21,22,23].

The calculated concentration of AgNPs in the colloidal solution was found to be 1.96 × 10^−6^ mol/L, and this concentration was used for all biological tests.

### 2.4. Analytical Techniques for Characterization of the AgNPs

After NPs preparation, the solution was used for ATR, transmission electron microscopy (TEM), dynamic light scattering (DLS), and zeta potential.

The UV-Vis spectra were not included due to the overlapping of surface plasmon resonance of AgNPs with the UV-Vis spectra of fenugreek extracts.

#### 2.4.1. ATR Spectra

The ATR spectra were measured using a VERTEX 70 FT-IR spectrometer (Bruker Optics, Ettlingen, Germany). The spectra were compared to the extract spectra and AgNPs’ spectra.

The conditions under which the spectra were measured are as follows:

- The instrument was equipped with an attenuated total reflection (ATR) accessory (PIKE MIRacle™ Single Reflection ATR device, ZnSe crystal, Madison, WI, USA) with detector—RT-DLaTGS and a beamsplitter of KBr.
- The spectra were analyzed with the OPUS-Spectroscopy Software, Bruker (Version 6.5, Bruker, Ettlingen, Germany).
- The spectra were collected in the range of 4000 cm^−1^ to 600 cm^−1^ with a resolution of 2 cm^−1^. The instrument and the optical parameters are: 25 scans for the background and for the samples, 5 mm aperture, and 10 kHz scanner velocity. Volumes of 50 μL from each sample were used.
- All spectra are presented in Transmittance (ordinate 0–100%) vs. wavenumber in cm^−1^ (abscissa).

#### 2.4.2. TEM

The TEM micrographs were registered by means of the Orius SC200D Model 833 CCD camera (Gatan Inc., Pleasanton, CA, USA) of the JEOL JEM 2100 HRTEM microscope (JEOL Ltd., Tokyo, Japan) at an accelerating voltage of 200 kV. In the preliminary preparation of TEM samples, the initial nanoparticle suspensions were dropped onto standard copper grids covered by amorphous carbon films and then dried under ambient conditions.

#### 2.4.3. DLS and Zeta Potential

The hydrodynamic diameter (d_h_), polydispersity index (PDI), and zeta potential of NPs prepared in water or ethanol (%) plant extracts were evaluated by dynamic light scattering (DLS) using a Zetasizer Nano ZS instrument (Malvern Instruments, Malvern, UK) equipped with a 633 nm laser. Measurements were carried out in triplicate at 37 °C and a scattering angle of 173°.

The mean hydrodynamic diameter (d_h_ ± SD) of the vesicles was calculated according to the Stokes–Einstein equation:

$$d_{h}=\frac{kT}{3\pi \eta D}$$

where (k) is the Boltzmann constant, (T) represents the absolute temperature (K), (η) corresponds to the viscosity of the dispersion medium at the measurement temperature, and (D) is the translational diffusion coefficient.

The ζ-potential of the vesicles was determined by electrophoretic light scattering using the same instrument under identical experimental conditions. The ζ-potential values were calculated based on the Smoluchowski equation:

ζ = 4πηυ/ε

where (η) denotes the viscosity of the solvent, (***ε***) is the electrophoretic mobility, and (υ) represents the dielectric constant of the medium.

### 2.5. Spasmolytic Activity Assesment

Initially prepared ethanol extracts were evaporated, and the dry residue was dissolved in pure DMSO (2 mL). The tested extracts were added in volumes not exceeding 150 μL to the 15 mL organ bath in order to preserve the physicochemical properties and physiological composition of the Krebs solution throughout the experiments [24].

#### 2.5.1. Animals and Ethical Approval

The study was performed using male Wistar rats (3–4 months old) obtained from the Medical University of Plovdiv’s experimental animal facility. Animals were housed under standard laboratory conditions at 22 ± 2 °C under a 12 h light/dark cycle, with free access to food and water.

Firstly, the animals were deeply anesthetized following established ethical guidelines for the humane treatment of experimental animals. Right after that, all gastric smooth muscle tissue was immediately isolated for ex vivo experiments.

All procedures involving animals were approved by the Institutional Animal Ethics Committee and were carried out in accordance with the principles established in European Directive 2010/63/EU concerning the protection of animals used for scientific purposes. In addition, official permits were obtained in accordance with the Bulgarian National and Academic Procedures: Statement No. 316 from 23 May 2024 of the Ethics Committee at the Medical University of Plovdiv, Bulgaria, as well as by the Animal Health and Welfare Directorate of the Bulgarian Food Safety Agency, Permit No. 400 valid through 6 June 2029.

#### 2.5.2. Ex Vivo Functional Assessment of Gastric Smooth Muscle Contractility

Circular gastric smooth muscle strips (11.0–12.5 mm long and 1.1–1.2 mm wide) were prepared from the isolated stomachs for ex vivo contractility experiments. In each experimental series, eight independent tissue preparations were used (*n* = 8), with each preparation representing one experimental replicate. The total number of experimental animals used was 16.

Each of the tissue preparations was placed vertically in a 15 mL tissue bath filled with Krebs physiological solution, heated up to 37 °C and continuously oxygenated with a carbogen mixture (95% O_2_ and 5% CO_2_). One end of each smooth muscle strip was attached to a stationary glass hook, while the other end was tied to an isometric force transducer (ADInstruments MLT0402, Dunedin, New Zealand) incorporated into a Radnoti 8-Unit Tissue Organ Bath System (Radnoti, Dublin, Ireland). Following mounting, the tissues were allowed to equilibrate for 60 min under resting conditions, with replacement of the Krebs solution at 15 min intervals to maintain tissue viability.

Mechanical activity was continuously recorded using the PowerLab Data Acquisition System coupled with LabChart software version 8.1.16 and the integrated Dose Response analysis module (ADInstruments, Dunedin, New Zealand). The functional integrity of the smooth muscle preparations was verified during the equilibration period by two consecutive applications of acetylcholine (10^−6^ M; Sigma-Aldrich, Germany), each of which elicited reproducible contractile responses with an average force around 5 mN.

The pharmacodynamic effects of aqueous and ethanolic fenugreek extracts, their corresponding AgNPs formulations prepared via green synthesis using the same extracts, were evaluated by cumulative concentration-dependent administration. Changes in mechanical activity were quantified as force generation and expressed in milliNewtons (mN).

Several functional parameters were analyzed throughout the experimental protocol, including contractile amplitude (mN), frequency of spontaneous contractions (contractions/min), tonic variations relative to basal spontaneous activity, and maximal contractile response (Emax) derived from cumulative concentration–response curves. These variables were subsequently used for comparative pharmacological characterization of the spasmogenic and spasmolytic effects of the investigated preparations.

### 2.6. Microbiological Tests

#### 2.6.1. Tested Microorganisms

To determine the antimicrobial activity of aqueous and ethanol extracts, the following test microorganisms were used: nine pathogenic microorganisms (*Staphylococcus aureus* ATCC 25923, *Listeria monocytogenes* NBIMCC 8632, *Klebsiella pneumoniae* ATCC 13883, *Enterococcus faecalis* ATCC 29212, *Escherichia coli* ATCC 25922, *Salmonella enteritidis* ATCC 13076, *Proteus vulgaris* ATCC 6380, *Pseudomonas aeruginosa* ATCC 9027 and *Candida albicans* NBIMCC 74, a spore-forming microorganism *Bacillus subtilis* ATCC 6633, four fungi (*Aspergillus niger* ATCC 1015, *Aspergillus flavus*, *Penicillium chrysogenum*, *Mucor* sp. and *Phizopus* sp.) and yeast (*Saccharomyces cerevisiae* ATCC 9763) from the collection of the Department of Microbiology at the University of Food Technologies, Plovdiv, Bulgaria, were selected for the antimicrobial activity test. Strains develop as follows: the yeast *S. cerevisiae* was cultured on MEA at 30 °C for 24 h. The fungi *A. niger*, *A. flavus*, *P. Chrysogenum* and *Mucor* sp. were grown on MEA at 30 °C for 7 days or until sporulation. *B. subtilis* was cultivated on Luria–Bertani agar with glucose (LBG agar) at 30 °C for 24 h. *S. aureus*, *L. monocytogenes*, *K. pneumoniae*, *E. faecalis*, *E. coli*, *S. enteritidis*, *P. Vulgaris*, *P. aeruginosa*, and *C. albicans* were cultured on LBG agar at 37 °C for 24 h.

Initially prepared ethanol extracts were evaporated, and the dry residue was dissolved in methanol.

#### 2.6.2. Culture Media

Luria–Bertani agar medium supplemented with glucose (LBG agar).

Composition (g/L): tryptone—10.0; yeast extract—5.0; NaCl—10.0; glucose—10.0 and agar—15.0. pH 7.5 ± 0.2. Sterilization: 121 °C/20 min.

Malt extract agar (MEA)

Composition (g/L): malt extract—30.0; mycological peptone—5.0; agar—15.0. pH 5.4 ± 0.2. Sterilization: 115 °C/10 min.

High antimicrobial activity is reported for inhibition zones 18 mm or more; moderate inhibition zones are between 12 and 18 mm; low inhibition zones are up to 12 mm, or completely absent inhibition zones.

#### 2.6.3. Antimicrobial Activity Assay 

Initially prepared ethanol extracts were evaporated, and the dry residue was dissolved in methanol. The antimicrobial activity of aqueous and methanolic extracts was determined by the agar-diffusion well method [25,26]. An amount of 18 mL of pre-melted LBG-agar medium was cooled to 40–45 °C and infected with the specified test microorganism (1.0 × 10^6^ cfu/ mL for spores of mold fungi and 1.0 × 10^8^ cfu/mL for viable cells of bacteria and yeast). LBG-agar medium is poured into Petri dishes (d = 9 cm), placed on a level surface. After pouring the infected culture medium, the Petri dishes were left for 1 h to solidify the agar. Using a cylindrical well puncher, 6 wells (d = 6 mm) were cut in the agar, and 60 μL each of the aqueous and methanolic extracts of each sample were analyzed in triplicate. The Petri dishes were thermostated at different temperature conditions (depending on the type of test—microorganism) for 24/48 h. The presence and degree of antimicrobial activity were determined by measuring the diameter of the inhibition zones around the agar wells.

### 2.7. Statistical Analysis

The statistical analysis was performed using SPSS 23.0 software (SPSS Inc., Chicago, IL, USA). All experimental data were presented as mean ± SD (standard deviation). Statistically significant differences for multiple comparisons were performed using Duncan’s test (chemical content).

Statistical significance between two independent groups (antimicrobial and spasmolytic activity) was analyzed by an independent sample Student’s *t*-test, and differences were considered significant at *p* < 0.05.

## 3. Results and Discussion

Fenugreek is one of the oldest medicinal plants and has traditionally been used for the treatment of various gastrointestinal disorders [27]. Fenugreek seeds are used for dysmenorrhea, dyspepsia, colic, diarrhea, gastritis, constipation, etc., [28,29,30,31]. Furthermore, fenugreek has been described as a central nervous system stimulant and a memory-enhancing agent [32].

Despite the broad spectrum of described pharmacological effects, information regarding the activity of fenugreek on isolated smooth muscle remains limited and incomplete [33,34]. Given the fact that acetylcholinesterase activity was found to be inhibited by methanolic extracts of fenugreek seeds [34] and acetylcholine is associated with gastrointestinal disorders, we set out to determine the effect of fenugreek seed extracts and AgNPs obtained from these extracts on smooth muscles.

Four types of fenugreek seeds, cultivated in Bulgaria (BG-1), India (BioBock, IN-1), and Egypt (FutuNatura, EG-1 and MyChef, EG-2), with varying soil types and climate conditions, were chosen for the study. The samples were bought from the Bulgarian commercial market and packaged in accordance with the manufacturer’s specifications. After selecting the plant material, the second task was to study the influence of the solvent during extraction. Water is a polar solvent used in eco-friendly extraction techniques. It is mostly used to extract water-soluble substances, such as phenolic compounds, anthocyanins, polysaccharides, saponins, vitamins, and minerals.

Ethanol, on the other hand, is an excellent solvent that can extract a wide range of lipophilic and hydrophilic components. It is known from the literature that plant extracts with a high content of polyphenols have the ability to modulate the reaction rate during the preparation of silver nanoparticles and are of crucial importance for determining the size of the final nanoparticles. It is also known that flavonoids have the ability to adsorb on the surface of NPs, changing their characteristics for a number of applications [35]. Rodríguez et al. indicated that the best solvent for the extraction of flavonoids is a water-ethanolic solution [36]. Since water and water-ethanolic extraction most often isolate carbohydrates, polyphenols, flavonoids, and water-soluble compounds that can help the reduction in Ag^+^, water and 80% ethanol were chosen as solvents. Our goal was to test whether the two solvents and the extracts obtained from them would have an impact on the size and shape of the resulting NPs, and consequently on their biological activity. Such a comparative study on the influence of geographic origin on fenugreek seeds and the NPs derived from them, using different types of extracts, was not found in the literature. The four batches of fenugreek seeds were ground, weighed, and submerged in water, resp. 80% ethanol. A hydromodule of 1:10 was chosen for the optimal extraction of the active ingredients. The next task in solving our goal was to obtain the NPs. AgNPs were chosen due to their established biological effects [16,37,38,39]. To synthesize AgNPs a standard protocol was used [17,18]. Following preparation, a variety of analytical methods, including ATR, FT-IR; dynamic light scattering (DLS); transmission electron microscopy (TEM); and zeta potential, were used to characterize the NPs.

### 3.1. Characterization of AgNPs

#### 3.1.1. FT-IR

**Figure 3:**
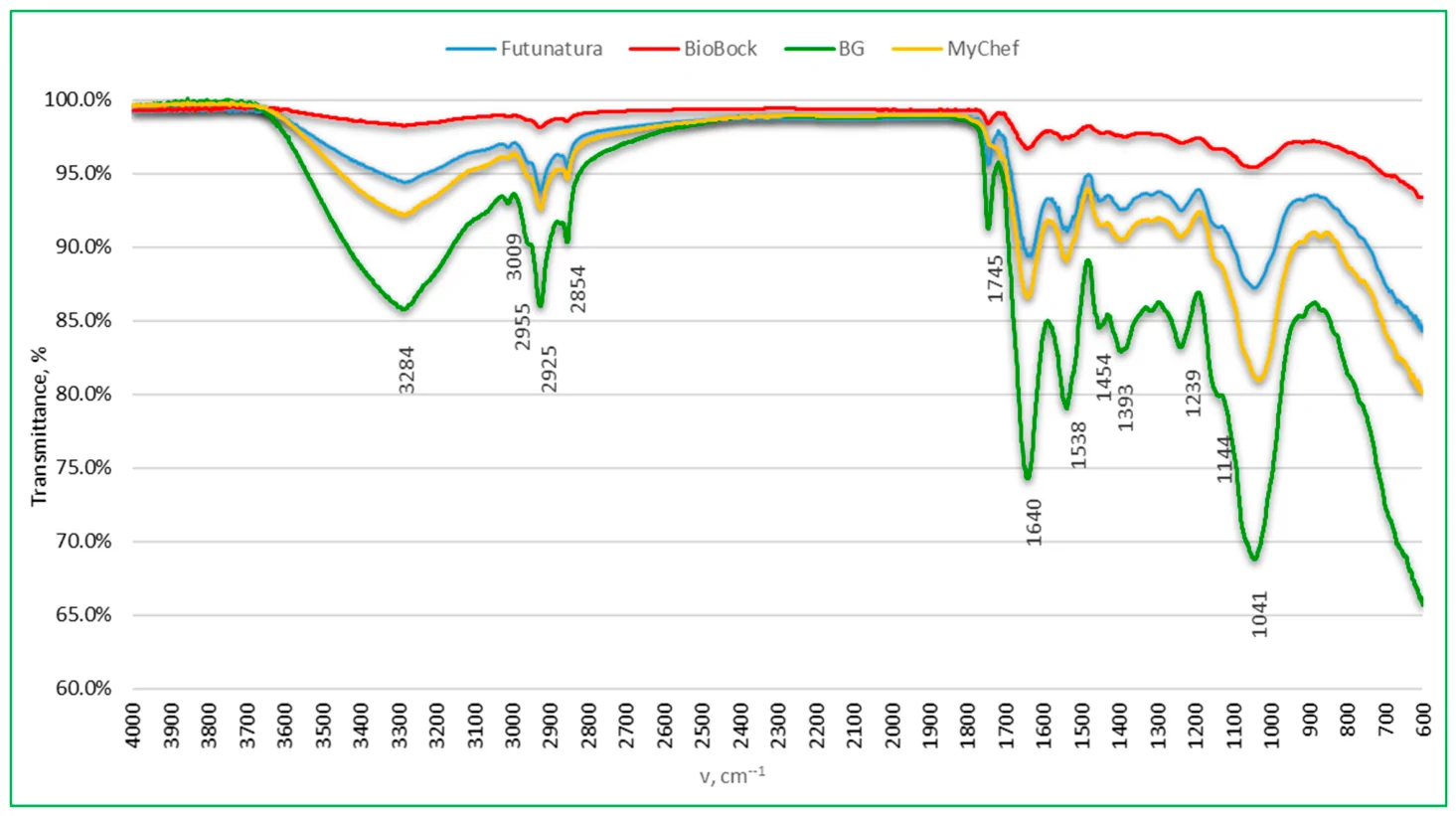
ATR-FT-IR spectra of fenugreek seeds (EG-1), IN-1, BG-1 and EG-2.

The dried seeds were finely ground, and then their FT-IR spectra were measured (Figure 3).

The latter are measured under the same conditions as all other samples described above. As the analysis of the spectra shows, it is obvious that a strong similarity was observed between them in the ranges in which the spectral bands corresponding to the following types of vibrations appear: around 3284 cm^−1^, assigned as N-H stretching vibrations (amide A protein), 1640 cm^−1^ (C=O, amide I), 1539 cm^−1^ (N-H bending vibration, amide II) and 1239 cm^−1^ (N-H bending, amide III). In both regions above and below 3000 cm^−1^, characteristic bands of –C-H vibrations in unsaturated and saturated bonds appear, respectively. The band at 1745 cm^−1^ is attributed to the >C=O vibration in other groups of compounds, such as carbonyl derivatives. This band appears as a shoulder in the spectrum of EG-2 and with the highest intensity in the spectrum of BG-1. The band around 1140 cm^–1^ appears as a shoulder related to the -C–O stretching vibrations, and an absorption band at 1041 cm^–1^, which is typical of starch. These bands are also characteristic of cellulose materials, such as fibers [40]. Differences in the intensities of the presented spectra were observed, most likely due to different quantitative levels of the target components in the respective plants that absorb in the mid-infrared region. Differences in the intensity of the indicated bands were observed comparing the spectra. We found that the highest intensity bands were characteristic of the Bulgarian type of sample, followed by the Egyptian sample (EG-2), and the least intensive were the Indian samples (IN-1). This is most likely due to the content of the target components in the fenugreek seeds from different countries.

**Figure 4:**
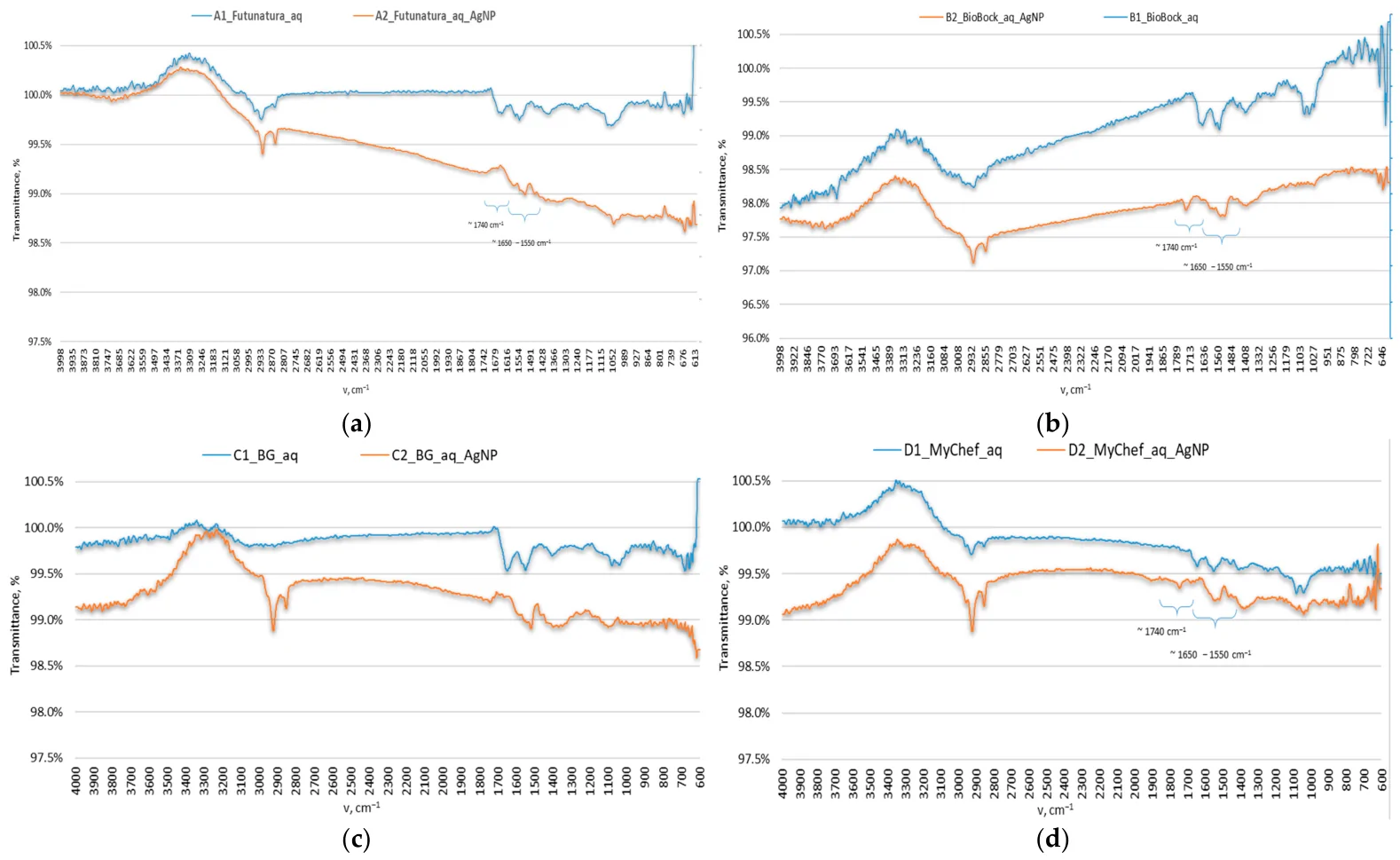
Comparison of spectra of (**a**) EG-1 fenugreek seeds aqueous extract_(blue) (A1) and AgNPs obtained from EG-1_fenugreek seeds aqueous extract (orange) (A2); (**b**) IN-1 fenugreek seeds aqueous extract_(blue) (B1) and AgNPs obtained from IN-1_fenugreek seeds aqueous extract (orange) (B2); (**c**) BG-1 fenugreek seeds aqueous extract_(blue) (C1) and AgNPs obtained from EG-2_fenugreek seeds aqueous extract (orange) (C2); (**d**) EG-2 fenugreek seeds aqueous extract_(blue) (D1) and AgNPs obtained from BG-1_fenugreek seeds aqueous extract (orange) (D2).

The analysis of the ATR spectra of the aqueous and ethanolic seed extracts and the AgNPs obtained revealed the presence of various characteristic peaks (Figure 4). When comparing the samples according to the solvent used for the extraction, compared to AgNPs obtained from this extract, we can see the following differences.

**Figure 5:**
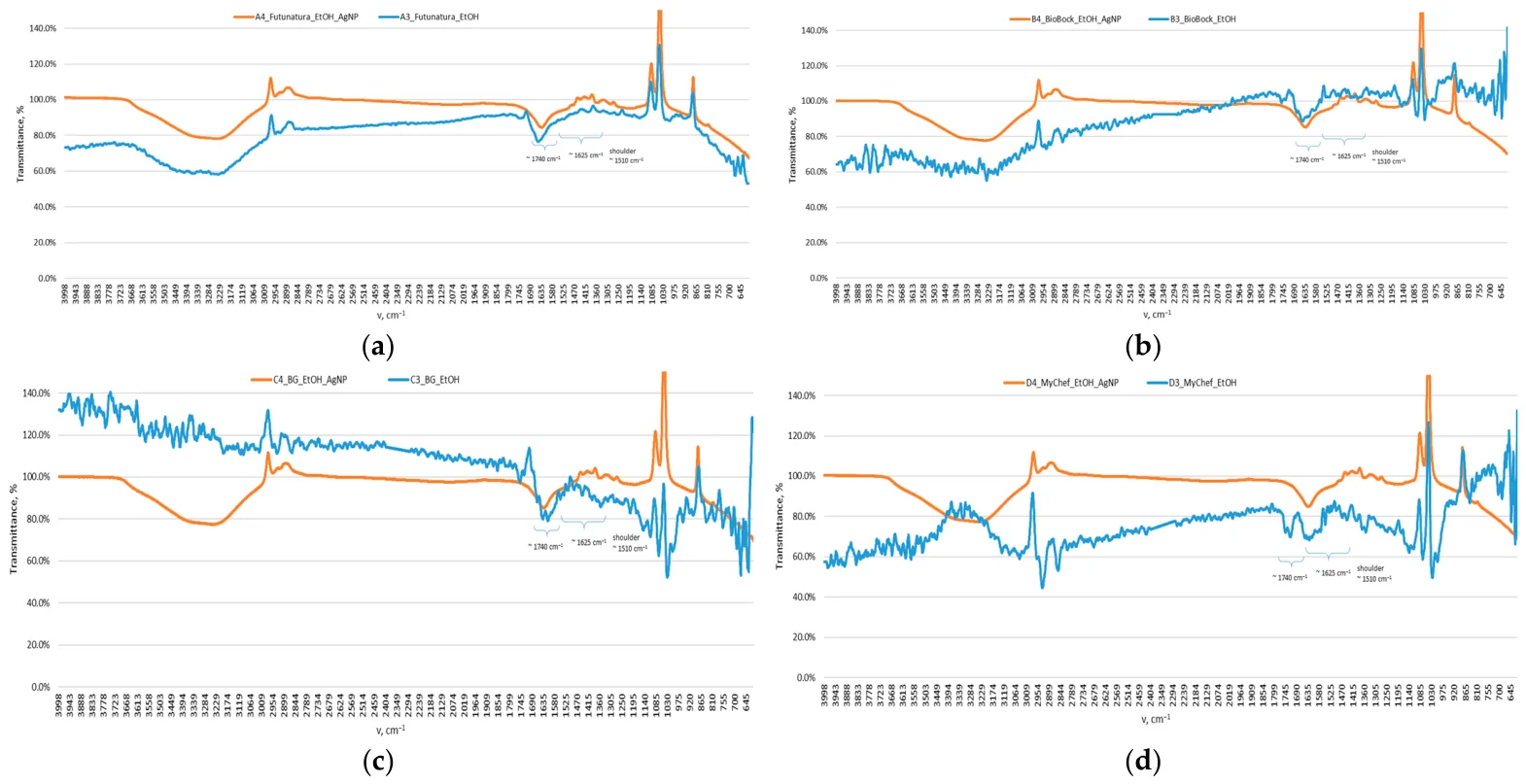
Comparison of spectra of (**a**) EG-1 fenugreek seeds ethanolic extract_(blue) (A1) and AgNPs obtained from EG-1_fenugreek seeds ethanolic extract (orange) (A2); (**b**) IN-1 fenugreek seeds ethanolic extract_(blue) (B1) and AgNPs obtained from IN-1_fenugreek seeds ethanolic extract (orange) (B2); (**c**) BG-1 fenugreek seeds ethanolic extract_(blue) (C1) and AgNPs obtained from EG-2_fenugreek seeds ethanolic extract (orange) (C2); (**d**) EG-2 fenugreek seeds ethanolic extract_(blue) (D1) and AgNPs obtained from BG-1_fenugreek seeds ethanolic extract (orange) (D2).

The FT-IR spectra of four distinct samples derived from aqueous fenugreek extracts and ethanol extracts, compared to AgNPs obtained from these extracts, are shown in Figure 4 and Figure 5. The null function was used to measure the spectra of all extracts in comparison to the solvent spectrum, which causes the negative peaks in some spectral regions. The spectrum analysis revealed that different solvents result in varying levels of extraction from the batch. It is clear from comparing the spectra of the fenugreek seeds (Figure 3) that bands in the range of 3000–2800 cm^−1^ during the aqueous extraction are associated with stretching vibrations of C-H bonds in alkyl groups, approximately 1640 cm^−1^ (C=O, amide I) and 1539 cm^−1^ (N-H bending vibration, amide II). Additionally, the ethanol extracts displayed bands at 1740 cm^−1^ (C=O, carbonyl) and approximately 1650 cm^−1^ (C=O, amide I), as well as a band at 1550 cm^−1^ that resembled a shoulder to 1650 cm^−1^.

Figure 4 presents the spectra of fenugreek aqueous extracts compared to the spectra of AgNPs obtained from these extracts. The observed differences between the spectra of samples are in two regions. Around 1740 cm^−1^, where the stretching vibration band for the >C=O group appears, which is rather reduced in the spectrum of the extract, but is present as a weakly intense band in the spectrum of AgNPs. In the interval 1650–1550 cm^−1^, it is seen that in the spectra of AgNPs compared to the extracts, the bands at 1640 cm^−1^ (C=O, amide I) and 1537 cm^−1^ (N-H bending vibration, amide II) are shifted to lower frequencies, as follows—around 1580 cm^−1^ and 1520 cm^−1^, respectively.

Figure 5 presents a comparison between spectra of ethanolic extracts and AgNPs obtained from these extracts. The observed differences between the spectra are approximately in the same regions as indicated in Figure 4. Around 1740 cm^−1^, where the stretching vibration band for the >C=O group appears, which is rather reduced in the spectrum of AgNPs, but is present as a weakly intense bulge in the spectrum of the extracts. The band at 1625 cm^−1^ is much more intense than the band around 1520 cm^−1,^ which has appeared as a shoulder to the band at 1625 cm^−1^.

In general, phenolic compounds (O–H bands) are thought to function as reducing agents that help to convert Ag^+^ to Ag^0^; whereas, carbonyl (C=O groups), proteins (COOH groups), and polysaccharide-associated groups (C–O) are suggested to help stabilize NPs and capping by surface adsorption and coordination.

We found that the samples from different geographical locations appear to have similar FT-IR spectra due to the presence of similar functional groups in the extracts. As a result, we can conclude that fluctuations in the relative quantities and ratios of bioactive molecules (such as phenols, proteins, and polysaccharides) as well as minor variations in extract composition could be the reason of variations in NPs’ size. These changes may have an impact on the nucleation and growth processes, which could ultimately result in variances in the size and characteristics of NPs.

To establish the size and shape of the AgNPs obtained, HRTEM and Zeta-potential were used.

#### 3.1.2. TEM Micrographs

The morphology, size distribution, and crystalline nature of AgNPs synthesized via *Fenugreek* (*Trigonella foenum-graecum*) extracts were characterized by means of TEM in Bright Field (BF TEM) and High Resolution (HR TEM) modes. A comparative study between the aqueous and ethanolic extraction media revealed significant differences in the nucleation and stabilization kinetics of the resulting nanostructures.

**Figure 6:**
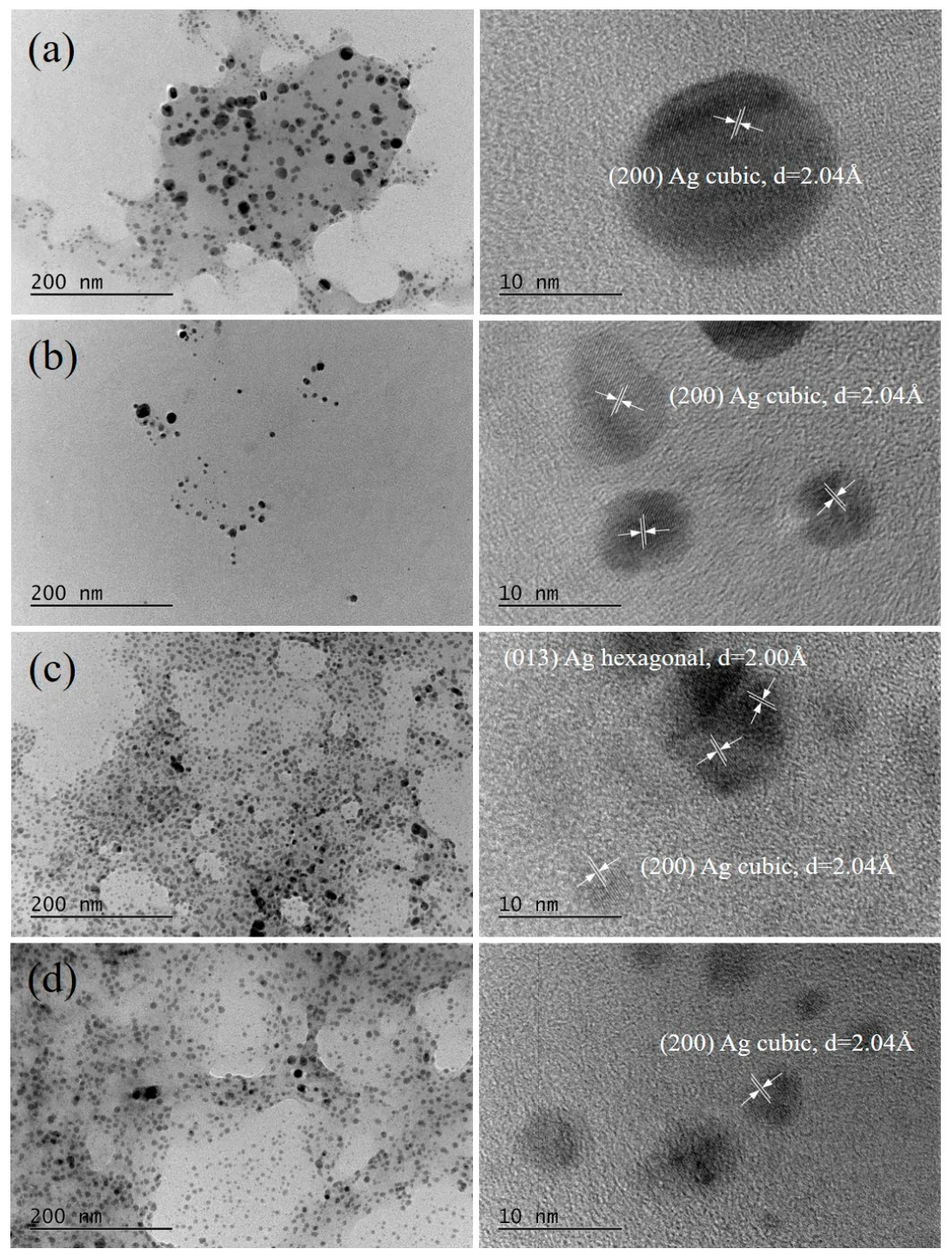
BF TEM micrographs (left images) and the corresponding HRTEM micrographs (right images) of AgNPs obtained from fenugreek seeds’ aqueous extracts: (**a**) IN-1 (BioBock, India), (**b**) EG-1 (FutuNatura, Egypt), (**c**) EG-2 (MyChef, Egypt), and (**d**) BG-1 (Bulgarian type).

The morphology of AgNPs obtained from the aqueous extract of all four types of fenugreek seeds is presented in Figure 6. The NPs are embedded within a prominent, low-contrast amorphous matrix, which corresponds to the high-molecular-weight phytochemicals (polysaccharides and proteins) typically found in water extracts [41]. The particles are predominantly spherical, which is typical for aqueous plant-mediated synthesis at neutral pH. TEM micrographs in all samples were acquired at an identical nominal magnification regime of 40 k and with a 200 nm scale bar, allowing direct visual and statistical comparison of nucleation, growth, and stabilization of the AgNPs.

**Figure 7:**
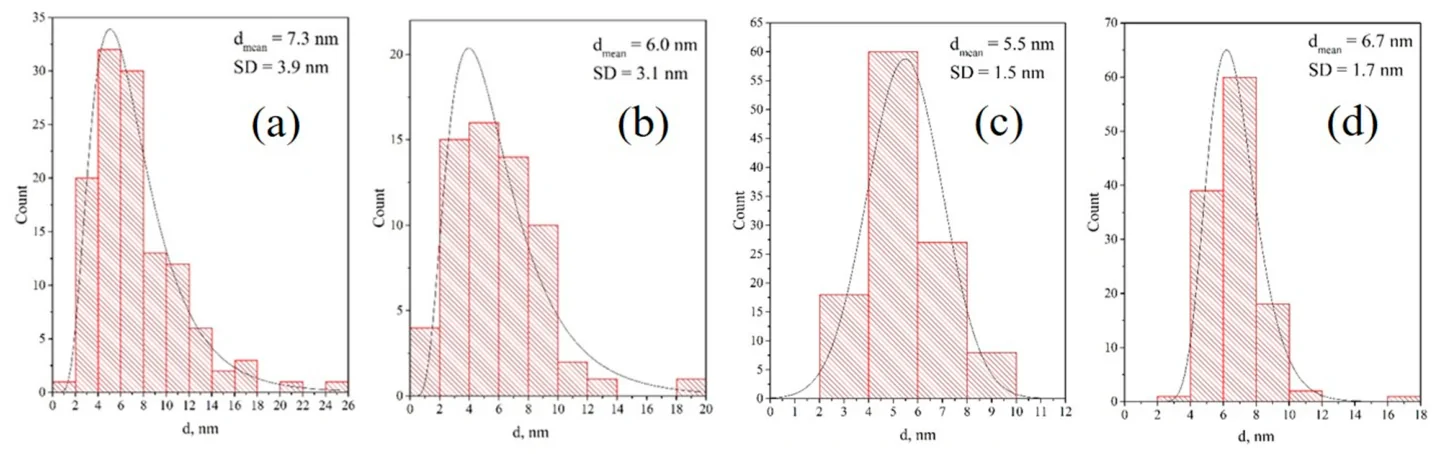
Size-distribution histograms of AgNPs from fenugreek aqueous extracts: (**a**) IN-1 (BioBock, India), (**b**) EG-1 (FutuNatura, Egypt), (**c**) EG-2 (MyChef, Egypt), and (**d**) BG-1 (Bulgarian type).

The size distribution of AgNPs synthesized from IN-1’s fenugreek aqueous extract (Figure 6a) shows polydispersity, with an average size of 7.3 nm (Figure 7a). A few larger particles or agglomerates are present, approaching up to 25 nm. The individual and well-separated particles observed in the aqueous extract suggest a significant steric stabilization, preventing direct metal-to-metal interaction even in high-density regions. This fact is evidence that the water-soluble phytochemicals act as highly efficient capping agents [41,42,43].

AgNPs mean diameter for those produced from EG-1’s fenugreek aqueous extract, on the other hand, is 6 nm (Figure 6b and Figure 7b). Notably, the particles exhibit spatial separation, suggesting a robust capping mechanism provided by the hydrophilic phytochemicals such as proteins, polysaccharides, and organic acids, prevalent in the aqueous extract. The background of the TEM micrograph of the water extract sample is relatively clear, indicating a high degree of metal precursor conversion and a lower concentration of non-participating organic residues on the carbon film.

The AgNPs synthesized from EG-2 fenugreek aqueous extract exhibited an average size of 5.5 nm (Figure 6c and Figure 7c). The presence of dense NP clusters indicated stronger interparticle interactions and comparatively lower colloidal stability. Particle assembly overlapped in certain areas, and the particles seemed less distributed overall. This aggregation may be attributed to insufficient capping efficiency or the presence of hydrophilic biomolecules dissolved in water, which may provide weaker steric stabilization during nanoparticle formation.

Finally, the micrograph of the AgNPs obtained from aqueous extract of BG-1 reveals a highly controlled nucleation event. The vast majority of the metallic cores reside at 6.7 nm (Figure 6d and Figure 7d). There are a few bigger particles, or agglomerates, with average size up to 25 nm in size. The AgNPs exhibit isotropic morphology, remaining strictly spherical or quasi-spherical. Under green synthesis conditions, spherical geometry represents the lowest surface-energy state, indicating that the capping agents in the water extract unselectively passivate all crystalline facets. It is also evident that, despite being tightly packed across the carbon grid support, individual NPs remain separated by a visible nanometer-scale gap. This distance is maintained by a faint, light-gray organic capping layer visible in the background, which provides a powerful steric and electrostatic barrier preventing silver NPs-to-silver NPs contact.

The phase composition of the nanoparticles synthesized by fenugreek aqueous extracts was examined by means of the HRTEM mode. The measured interplanar distances, compared with the data included in the Crystallography Open Database (COD), unambiguously prove that the synthesized nanoparticles are silver. In most samples, the presence of cubic silver is demonstrated, and in the case of EG-2, a co-existence of the cubic and hexagonal silver is established.

The size distribution of aqueous extracts is less than 10 nm, according to the histogram shown in Figure 7. For many chemical or environmentally friendly synthesis techniques, tiny AgNPs (<10 nm) predominate. The low polydispersity and limited size distribution point to regulated nucleation and growth conditions during synthesis. Larger particles (outliers) may indicate subsequent nucleation or mild agglomeration. These distributions are common for wet-chemical reduction preparation procedures, where growth dynamics result in a primary population with a few bigger crystals accumulating over time—in this example, up to 25 nm. The most prevalent particle size range is indicated by the peak population, which is located between 4 and 8 nm. The calculated mean diameter of the AgNPs in this study are as follows: IN-1 with average size of 7.3 nm; EG-1 with average size of 6 nm; EG-2 with average size of 5.5 nm; and BG-1 with average size of 6.7 nm.

Ethanol, on the other hand, extracts biologically active organic reducing compounds, such as polyphenols, flavonoids, tannins, carotenoids, and chlorophylls. Ag^+^ is reduced to Ag^0^ more quickly when these compounds are isolated, which may speed up the kinetics of nanoparticle formation.

**Figure 8:**
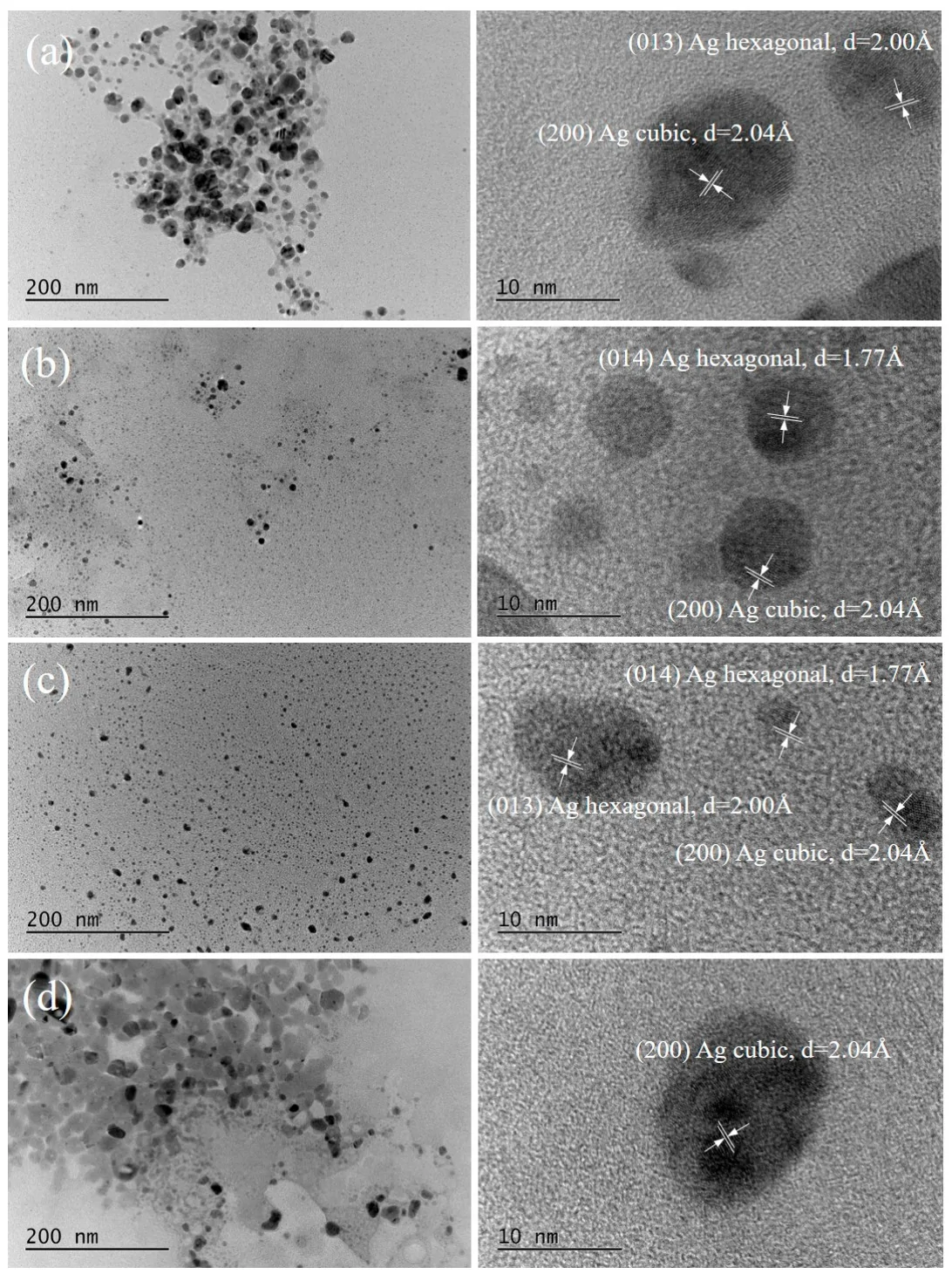
BF TEM micrographs (left images) and the corresponding HRTEM micrographs (right images) of AgNPs obtained from fenugreek seeds’ ethanolic extracts: (**a**) IN-1 (India), (**b**) EG-1 (FutuNatura, Egypt), (**c**) EG-2 (MyChef, Egypt), (**d**) BG-1 (Bulgarian type).

**Figure 9:**
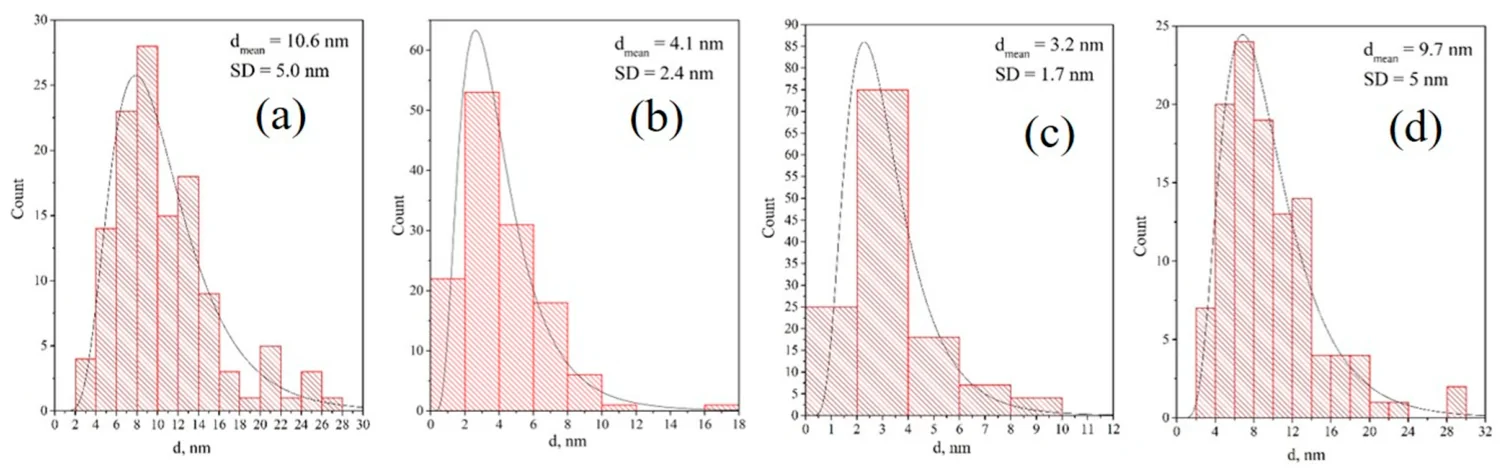
Size-distribution histograms of AgNPs from fenugreek ethanolic extracts: (**a**) IN-1 (BioBock, India), (**b**) EG-1 (FutuNatura, Egypt), (**c**) EG-2 (MyChef, Egypt), and (**d**) BG-1 (Bulgarian type).

For example, the AgNPs synthesized from the IN-1’s fenugreek ethanolic extract have larger sizes compared to the AgNPs from aqueous extract’s, with average size of 10.6 nm (Figure 8a and Figure 9a). A defining feature of this sample is the high frequency of crystalline defects within the individual particles. While the AgNPs remain nearly spherical, they exhibit more defined facets compared to the aqueous sample. This indicates that the ethanol-soluble compounds may selectively bind to certain crystallographic planes during growth. These particles also show a higher tendency for agglomeration. The organic scaffold appears more compact and denser than the water extract. This is attributed to the presence of lower-polarity resins or lipids, which provide a thinner capping layer, leading to reduced inter-particle repulsion and closer packing during the drying process [44].

AgNPs obtained from ethanolic extracts of EG-1’s fenugreek demonstrate a significant shift toward ultrasmall dimensions. The population is characterized by a high electron density of so-called “seed-like” particles, with an average size of 4.1 nm (Figure 8b and Figure 9b).

AgNPs obtained from EG-2 fenugreek ethanolic extract showed improved dispersion and a more homogeneous distribution of the NPs across the TEM grid (Figure 8c and Figure 9c). These particles appeared smaller, with an average size of 3.2 nm and relatively more uniform in size with reduced agglomeration compared to the aqueous-extract-derived NPs. The enhanced stabilization in the ethanolic extract may be associated with the extraction of greater amounts of phenolic compounds, flavonoids, and other bioactive constituents soluble in ethanol, which can act as both reducing and effective capping agents during the synthesis.

Switching the extraction solvent to ethanol in BG-1 radically alters the growth kinetics of synthesized AgNPs. The size distribution becomes broad and polydisperse. We observe tiny seed particles with nearly elliptical shapes with average size of 9.7 nm directly adjacent to large, mature crystallites exceeding 50 nm (Figure 8d and Figure 9d). This is a classic indicator of secondary growth mechanisms, such as Ostwald ripening or oriented attachment, occurring during or after synthesis [44]. Here, we can see a shift to anisotropic crystallization. The particles display distinct and sharp geometric boundaries. This demonstrates that the compounds dissolved in the ethanolic extract bind selectively to specific crystal facets, allowing preferential growth along predetermined crystallographic axes. Also, the dispersion level drops considerably, showing extensive agglomeration. Large, multi-particle clusters dominate the grid. In several zones, the grain boundaries appear to fuse into interconnected networks. The organic biomolecules present in ethanol fail to form a continuous, protective envelope around the newly formed metallic nuclei, leading to localized agglomeration. TEM micrographs in all samples were acquired at an identical nominal magnification regime of 40 k and with a 200 nm scale bar, allowing direct visual and statistical comparison of nucleation, growth, and stabilization of the AgNPs.

The observed differences can be attributed to the nucleation-growth kinetic model [44]. Ethanolic extract likely induces rapid nucleation in AgNPs’ synthesis. The higher concentration of strong reducing phytochemicals in ethanol causes a fast consumption of silver ions, leading to a high density of small nuclei. Because the precursor is depleted quickly, the subsequent growth phase is limited, resulting in the observed small diameters, especially for Egypt samples EG-1 and EG-2.

The size distribution of ethanolic extracts is up to 10.6 nm, according to the histogram shown in Figure 9. The low polydispersity and limited size distribution point to regulated nucleation and growth conditions during synthesis. The calculated mean diameters of the AgNPs in this study are as follows: IN-1 with an average size of 10.6 nm; EG-1 with an average size of 4.1 nm; EG-2 with an average size of 3.2 nm; and BG-1 with an average size of 9.7 nm.

Overall, EG-1 and EG-2 in both solvents form smaller NPs with an average size between 3.2 and 6 nm, while IN-1 and BG-1 obtained bigger NPs with an average size between 6.7 and 10.6 nm.

#### 3.1.3. DLS and Zeta-Potential

**Figure 10:**
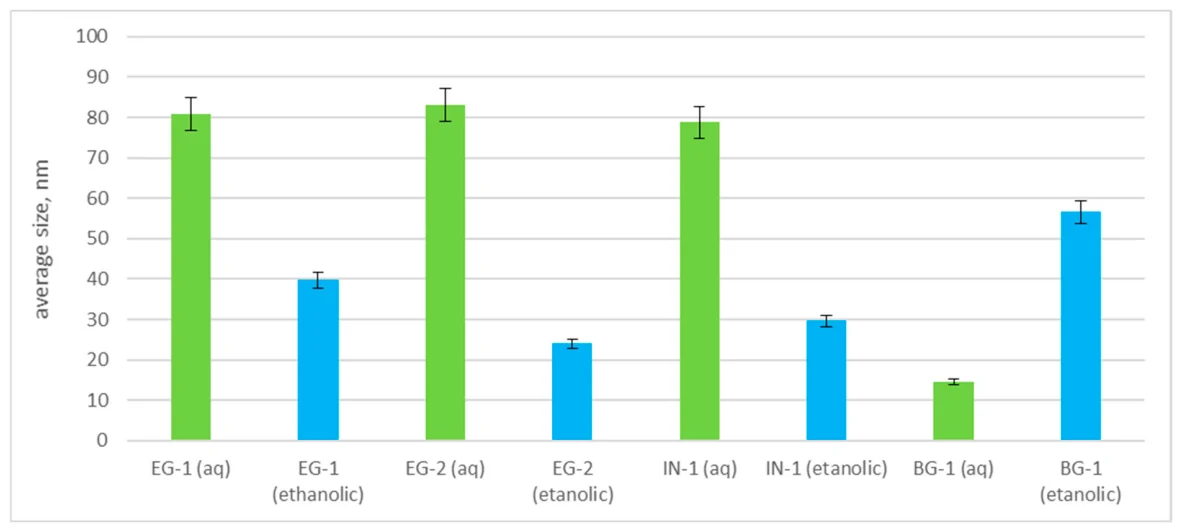
Size distribution of AgNPs synthesized using aqueous (green) and ethanolic (blue) extracts, obtained from DLS analysis of four fenugreek seeds: IN-1, EG-1, EG-2, and BG-1.

The DLS analysis showed that the physicochemical properties of the synthesized AgNPs strongly depended on both the type of plant extract and the extraction solvent (Table 1, Figure 10).

Based on the data obtained from DLS analysis, we can conclude that AgNPs synthesized using ethanolic extracts exhibited lower PDI values and more stable surface charges compared to NPs synthesized from aqueous extracts. Among all samples, the smallest NPs were obtained using the Bulgarian type BG-1 (aqueous) with an average size of 14.6 nm. The other three aqueous extracts produced bigger NPs. In general, aqueous extracts produced larger particles, with EG-2 showing the largest average size (83.1 nm), followed by EG-1 with an average size of 80.8 nm and IN-1 with an average size of 78.8 nm. Ethanolic extracts, on the other hand, produced smaller NPs with sizes in the range of 24–56.6 nm.

In general, using the DLS data, we can conclude that for three of the samples, aqueous extracts obtained bigger NPs compared to the ethanolic extracts, except for BG-1. We can attribute this data to the specific substances contained in the Bulgarian type of fenugreek.

Based on the obtained data, we can assume that aqueous extracts isolated more hydrophilic phytochemicals, polysaccharides, and proteins, which may encourage aggregation or less regulated nucleation during NPs formation, and therefore may be connected to the greater particle size. Ethanolic extracts, on the other hand, extracted more polyphenols, flavonoids, and other reducing substances that improve nucleation and stability and produce smaller, more homogeneous NPs. The obtained data is fully consistent with the literature [45,46,47].

The PDI values further supported these observations. Lower PDI values indicate a narrower particle size distribution and better homogeneity. For comparatively homogeneous nanoparticle dispersions, PDI values less than 0.3 are often regarded as acceptable. Ethanolic extract-derived NPs showed relatively low PDI values (0.23–0.30), suggesting a more uniform NPs population. In particular, Bulgarian fenugreek (aquesous) exhibited the lowest PDI (0.19 ± 0.01), indicating highly monodisperse particles. This data is confirmed by the DLS and TEM analysis.

One important physicochemical measurement that is frequently utilized as an indicator of colloidal stability is zeta-potential. It represents the surface charge of nanoparticles in suspension. Strong electrostatic repulsion prevents aggregation, therefore NPs with high absolute zeta potential values (≥±30 mV) are often regarded as highly stable [48]. Negative zeta-potential is frequently seen in plant-mediated synthesis of AgNPs [49,50]. The adsorption of anionic biomolecules found in plant extracts, including proteins, organic acids, polysaccharides, and phenolic compounds, is the source of this negative charge. These biomolecules generate a bio-organic cap surrounding the surface of the NPs by acting as capping, stabilizing, and reducing agents. The negative charge is observed due to COO- of proteins, oxidation of saccharides to aldonic acids, etc.

The majority of the obtained NPs suspensions had negative surface charges, which support colloidal stability and electrostatic repulsion, according to the ζ-potential values. Due to considerable electrostatic repulsion between particles, BG-1 (aqueous) exhibited the largest negative ζ-potential (−24 ± 0.64 mV), while EG-2 (aqueous) had an extremely low ζ-potential (−6 ± 0.42 mV), which is consistent with its high PDI value. NPs produced from ethanolic extract generally displayed moderate ζ-potential values between −26 and −31 mV (except EG-1 with ζ-potential −61 ± 0.28 mV), indicating rather stable dispersions.

Overall, the results demonstrate that most of the ethanolic extracts were more effective for producing smaller, more homogeneous, and relatively stable AgNPs compared to aqueous extracts. The differences observed among plant extracts may be attributed to variations in their phytochemical composition, which directly influence nanoparticle nucleation, growth, and stabilization.

The increase in size in the DLS measurements compared to TEM may be attributed to the fact, that the TEM analysis did not take into account any coating or stabilizing agent because it simply examined the metallic silver core’s average diameter. In contrast, the DLS analysis measured the dynamic fluctuation and velocity of particles in suspended clusters, which is the metallic silver core and the molecules that bind to the NPs’ surface. Depending on the structure, these particles may go through a process of solvation and expansion in the solution. As a result, the hydrodynamic diameter determined by DLS was greater than the size determined by TEM [45,51,52].

### 3.2. Spasmolytic Activity

Inhibition of spontaneous or induced contractile responses is a characteristic of antispasmodic activity. A reduction in frequency, amplitude, or baseline tonic tension are parameters commonly used to illustrate the spasmolytic effect. An increase in the frequency of contractions or an increase in tonic tension are indicators of spasmogenic activity, which leads to an improvement in smooth muscle contractility. The experimental approach was based on the hypothesis that NPs biosynthesis may substantially influence the biological activity of the plant extracts, as well as modify their contractile or relaxant effects on gastrointestinal smooth muscle tissue. Recent studies investigating the effects of fenugreek on different types of isolated smooth muscle are still lacking, as are sufficient data on its precise mechanisms of action, active compounds, and dose-dependent effects. The present study clearly demonstrates modulation of spontaneous gastric smooth muscle contractility by extracts of *Trigonella foenum-graecum*, with effects strongly dependent on the extraction medium and on the presence of biogenically synthesized AgNPs. All four tested aqueous extracts in the absence of AgNPs did not induce any statistically significant changes in contractile parameters, including amplitude, frequency, or tonic tension, compared with baseline spontaneous activity. When the same aqueous extracts were administered in combination with biogenic AgNPs, a marked potentiation of pharmacological activity was observed. All four extract–nanoparticle formulations exhibited pronounced modulation of smooth muscle contractility at identical working concentrations (Table 2). This was characterized by substantial alterations of spontaneous motility patterns and a clear enhancement of the functional response compared with the corresponding extracts applied alone. These findings indicate a strong synergistic or sensitizing effect of AgNPs incorporation on the bioactivity of the aqueous fenugreek extracts.

The results demonstrated that for all batches of fenugreek seeds (aqueous) or (ethanolic) extracts, they had only a negligible impact on contractile amplitude, while the equivalent AgNPs obtained from these aqueous extracts significantly increased the tonic tension response and contractile modulation.

This suggests a synergistic interaction between fenugreek-derived phytochemical compounds and the NPs. The obtained NPs potentially enhance tissue bioavailability, cellular uptake, or receptor-level interactions. Such enhancement may be associated with NP-induced alterations in membrane permeability and in modified ligand–receptor dynamics, as previously described for plant-mediated nanomaterials in biological systems [17,18]. The increased activity observed with ethanolic extracts in the present study may be attributed to the higher solubility of polyphenols, flavonoids, and other reducing compounds, which may contribute to smooth muscle modulation [53].

The intriguing result of this work is that, in comparison to aqueous-derived AgNPs, ethanol-derived AgNPs showed less spasmolytic effectiveness. This result implies that surface chemistry and the type of capping agents are important factors in determining biological activity, rather than nanoparticle size alone.

Among all tested extracts, the AgNPs (aqueous) obtained from Bulgarian fenugreek seeds exhibited the highest tonic tension response, indicating the strongest pharmacological activity under the applied experimental conditions. We can explain this data with the smallest size of this sample, based on the DLS results, as well as with the fact that ethanol extracts are rich in polyphenols and flavonoids and therefore have a great ability to adsorb onto the surface of nanoparticles. Although these substances are efficient reducing agents, their strong binding may result in a dense organic layer that produces steric hindrance and restricts AgNPs’ ability to interact with ion channels or smooth muscle receptors. The release of Ag^+^ ions, which are frequently linked to biological activity, may also be decreased by this surface passivation.

Aqueous extracts, on the other hand, are more likely to have larger concentrations of proteins and polysaccharides, which can form a more flexible and porous surface corona and serve as stabilizing factors. These biomolecules may operate as “biological adjuvants,” improving the local bioavailability of active compounds or promoting contact with biological membranes. We can confirm this data with the negative charge of zeta potential, made of carboxylate ions and amino acid residues from carbohydrates and proteins.

Variations in the kinetics of Ag^+^ ion release could be another important element. Aqueous-derived nanoparticles may provide more sustained or accessible Ag^+^ release, hence improving pharmacological efficacy, while ethanol-derived nanoparticles may show slower ion release due to stronger capping. Future research can evaluate these theories by comparing surface composition, measuring Ag^+^ release profiles, and assessing nanoparticle–cell interactions by cellular or receptor-based assays. When taken together, these factors demonstrate that size, surface chemistry, and capping biomolecules interact intricately to control the biological activity of green-synthesized AgNPs rather than just particle size.

The nature of the observed responses is consistent with earlier reports describing concentration-dependent and context-specific effects of *Trigonella foenum-graecum* on muscle performance [54]. Fenugreek seed extracts have been reported to exhibit both inhibitory and stimulatory effects on gastrointestinal motility depending on the extraction method, dose, and tissue model. More recent investigations have also highlighted a bidirectional pharmacological profile, ranging from relaxation to stimulation of contractile responses in isolated smooth muscle preparations [34].

In summary, our results support the hypothesis that the pharmacological profile of *Trigonella foenum-graecum* is not fixed but rather dynamically modulated by extraction methodology and nanostructure integration. The interaction between phytochemical constituents and AgNPs appears to shift the functional outcome toward enhanced smooth muscle reactivity, highlighting the potential of nano-based phytotherapy as a strategy for optimizing gastrointestinal pharmacological effects.

Fenugreek is well-known for its antimicrobial properties; therefore, its antimicrobial potential was assessed compared to AgNPs obtained from aqueous and ethanolic extracts.

### 3.3. Antimicrobial Activity

Silver has been valued for its antibacterial properties for a long time. Reducing silver to the nanoscale (1–100 nm) increases its surface area and improves its ability to penetrate bacteria. The exceptional antibacterial activity of AgNPs can be advantageous, particularly against pathogens resistant to traditional antimicrobials [55].

Evaluating the antimicrobial and antibacterial activity of AgNPs from fenugreek seed extracts allows for drawing a clearer picture of silver nanoparticles’ potential to target gut microbiota and inflammation in the gastrointestinal tract. For this purpose, initially prepared ethanol extracts were evaporated, and the dry residue was dissolved in pure methanol. There are no zones of inhibition in either of the two extracts of any of the fenugreek types, nor in the two controls (water and methanol) used as solvents for this analysis.

Based on the obtained data, AgNPs demonstrated promising antifungal activity against all tested fungal strains, *Aspergillus niger* (with an inhibition zone of 20–22 mm), *Penicillium chrysogenum* (with an inhibition zone of 18–21 mm), and moderate activity against *Rhizopus* spp. (inhibition zone of 12–16 mm)*, Mucor* spp. (inhibition zone of 12–15 mm), as well as yeast, *Saccharomyces cerevisiae* (with an inhibition zone of 13–18 mm) (Table 3). The most common spoilage fungi are *Aspergillus* spp. and *Penicillium* spp. They have the ability to produce mycotoxins, which are extremely harmful to both humans and animals [56,57]. *Aspergillus* species are the most prevalent, accounting for up to 22% of air spore samples [58]. *Aspergillus* infections mostly affect the respiratory system. In the presence of high humidity, oxygen, and carbon dioxide, *Aspergillus* spores can germinate in the lungs [59,60,61].

The most frequent species that causes aspergillosis is *Aspergillus* fumigatus, which is followed by *A. flavus* and *A. niger*. One of the most frequent infections that causes mycosis in humans is *A. niger*. Systemic invasive infections brought on by these molds can be fatal for at least 50% of affected people [62]. The recent increase in drug-resistant isolates of *Aspergillus* species has been associated with long-term exposure to antifungals [63].

*Penicillium* species, on the other hand, are saprophytes, plant pathogens, and human pathogens [64]. Industry has employed *P. chrysogenum* to produce the common antibiotic penicillin [65]. Conversely, necrotizing pneumonia has been associated with *Penicillium chrysogenum* [66]. There have also been reports of *Penicillium chrysogenum*-related acute respiratory failure that resulted in death [67].

AgNPs in solution may be able to stick to and saturate fungal hyphae, killing the fungus cells, according to recent research [68].

According to our findings, AgNPs significantly reduced the growth of the fungus *Aspergillus niger* and *P. chrysogenum*.

The AgNPs obtained from the Bulgarian type fenugreek BG-1 (ethanolic) was the most potent against *A. niger* (with an inhibition zone of 22 mm), while the aqueous extract from the same batch was moderately active against *Mucor* spp. (15 mm). The Egyptian fenugreek EG-1 (aqueous) is more effective against *Penicillium chrysogenum* (21 mm). The Indian fenugreek IN-1 is more potent against *Rhizopus* spp. (inhibition zone of 18 mm for ethanolic extract) and *Saccharomyces cerevisiae* (with an inhibition zone of 18 mm for both aqueous and ethanolic extracts). The obtained results were similar to those for AgNPs obtained from fenugreek leave extracts [69].

Based on their higher surface-area-to-volume ratio—which can enhance binding at different target sites, promote diffusion, and have a lower propensity to aggregate—smaller NPs are often shown to have more effective antifungal activity than larger NPs [70].

Various nanoparticles rapidly and stably associate with spores, without specific functionalization. Small nanoparticle size, as opposed to material, charge, or “stealth” alterations, increased the development of nanoparticle-spore complexes [71].

Due to their strong antifungal potential, especially the Bulgarian type BG-1 and the Egyptian EG-1 against *A. niger* and *Penicillium chrysogenum*, we can conclude that AgNPs obtained from fenugreek seed extracts hold a good promise as therapeutic candidates.

## 4. Conclusions

In summary, based on the obtained results, both the physicochemical properties and the biological activity of synthesized AgNPs from *Trigonella foenum-graecum* extracts are strongly influenced by the extraction solvent and geographic origin of the plant material.

Overall, the geographic origin significantly influences the nanoparticle morphology, particle size distribution, and aggregation behavior. The Egyptian fenugreek (both EG-1 and EG-2) formed smaller NPs in both solvents with an average size between 3.2 and 6 nm, while Indian and Bulgarian type obtained bigger NPs with an average size between 6.7 and 10.6 nm.

The majority of the obtained AgNPs had negative surface charges, which support colloidal stability and electrostatic repulsion.

From a biological point of view, fenugreek extracts from all samples exhibited a weak effect on gastrointestinal contractility, while AgNPs obtained from these extracts showed an increased spasmogenic effect. These results suggest synergy, which improves bioavailability and functional efficacy. Among all tested extracts, the AgNPs (aqueous) obtained from Bulgarian fenugreek seeds exhibited the highest tonic tension response, indicating the strongest pharmacological activity under the applied experimental conditions. We can explain these data with the smallest size of this sample (14.6 nm based on the DLS data).

The intriguing result of this work is that, in comparison to aqueous-derived AgNPs, ethanol-derived AgNPs showed less spasmolytic effectiveness. This result implies that surface chemistry, the type of capping agents, the type of the extracted bioactive molecules, surface composition, and the strength of the binding are important factors in determining biological activity, rather than nanoparticle size alone.

Future research can evaluate these theories by comparing surface composition, measuring Ag^+^ release profiles, and assessing nanoparticle–cell interactions by cellular or receptor-based assays. When taken together, these factors demonstrate that size, surface chemistry, and capping biomolecules interact intricately to control the biological activity of green-synthesized AgNPs rather than just particle size.

The synthesized AgNPs also demonstrated a promising antifungal activity against all tested fungal strains, *Aspergillus niger* (with an inhibition zone of 20–22 mm), *Penicillium chrysogenum* (with an inhibition zone of 18–21 mm), and moderate activity against *Rhizopus* spp. (inhibition zone of 12–16 mm)*, Mucor* spp. (inhibition zone of 12–15 mm), as well as yeast, *Saccharomyces cerevisiae* (with an inhibition zone of 13–18 mm). The most common spoilage fungi are *Aspergillus* spp. and *Penicillium* spp.

Future research will include long-term storage data, such as size changes and aggregation, and examine the relationship between these parameters, ζ potential, and spasmolytic activity.

The obtained results demonstrate that NPs showed a potent method for enhancing plant extracts’ biological potential, where geographical origin and extraction strategy can be used as controllable parameters to optimize biological performance.

## Figures and Tables

**Table 1:** Size, PDI, and zeta-potential, measured by DLS.

Sample	Size (nm)	PDI	ζ-Potential (mV)
AgNPs (aqueous) (EG-1)	80.8	0.34 ± 0.02	−23 ± 2.97
AgNPs (ethanolic) (EG-1)	39.8	0.23 ± 0.01	−61 ± 0.28
AgNPs (aqueous) (EG-2)	83.1	0.64 ± 0.14	−6 ± 0.42
AgNPs (ethanolic) (EG-2)	24.0	0.30 ± 0.05	−31 ± 1.34
AgNPs (aqueous) (IN-1)	78.8	0.41 ± 0.01	−19 ± 1.63
AgNPs (ethanolic) (IN-1)	29.6	0.24 ± 0.03	−26 ± 0.78
AgNPs (aqueous) (BG-1)	14.6	0.19 ± 0.01	−24 ± 0.64
AgNPs (ethanolic) (BG-1)	56.6	0.28 ± 0.03	−27 ± 0.53

**Table 2:** Comparative effects of aqueous and ethanolic fenugreek extracts and their corresponding AgNPs formulations on spontaneous gastric smooth muscle contractility on isolated rat stomach preparations. Data are expressed as mean ± SD. Statistical analysis was performed using Student’s *t*-test. *p* < 0.05. * Represents a statistically significant difference compared with baseline spontaneous activity. ND—no detectable effect.

		Contractile Activity Parameters		
Fenugreek Seed Batch	Extract Type	Contractile Amplitude (mN)	Frequency (Contractions/min)	Tonic Tension Response (Emax)
IN-1 (50 μL)	aqueous	1.78 ± 0.01	ND	ND
	AgNPs (aqueous)	1.72 ± 0.03	ND	3.27 ± 0.30 *
	ethanolic	1.75 ± 0.01	ND	ND
	AgNPs (ethanolic)	1.76 ± 0.04	ND	2.25 ± 0.06
EG-2 (50 μL)	aqueous	1.89 ± 0.05	ND	ND
	AgNPs (aqueous)	1.80 ± 0.04	ND	3.33 ± 0.32 *
	ethanolic	1.87 ± 0.04	ND	ND
	AgNPs (ethanolic)	1.81 ± 0.02	ND	2.18 ± 0.04
EG-1 (50 μL)	aqueous	1.77 ± 0.03	ND	ND
	AgNPs (aqueous)	1.98 ± 0.09	ND	3.24 ± 0.19 *
	ethanolic	1.77 ± 0.02	ND	ND
	AgNPs (ethanolic)	1.78 ± 0.01	ND	2.34 ± 0.08
BG-1 (50 μL)	aqueous	1.88 ± 0.05	ND	ND
	AgNPs (aqueous)	1.94 ± 0.07	ND	3.64 ± 0.39 *
	ethanolic	1.85 ± 0.04	ND	ND
	AgNPs (ethanolic)	1.89 ± 0.05	ND	2.27 ± 0.06
		baseline spontaneous activity		
		1.70 ± 0.02	6.0 ± 0.5	1.88 ± 0.07

**Table 3:** Antimicrobial activity of AgNPs, obtained from fenugreek extracts.

Tested Microorganisms	Inhibition Zone, mm							
	AgNPs, Obtained from Bulgarian Fenugreek (BG-1)		AgNPs, Obtained from Egyptian Fenugreek (EG-1)		AgNPs, Obtained from Egyptian Fenugreek (EG-2)		AgNPs, Obtained from Indian Fenugreek (IN-1)	
	Aqueous	Ethanolic	Aqueous	Ethanolic	Aqueous	Ethanolic	Aqueous	Ethanolic
*Staphylococcus aureus*	10 ± 0.0	9 ± 0.0	9 ± 0.0	9 ± 0.0	9 ± 0.0	9 ± 0.0	10 ± 0.0	10 ± 0.0
*Listeria monocytogenes*	10 ± 0.0	11 ± 0.0	9 ± 0.0	11 ± 0.0	10 ± 0.0	10 ± 0.0	10 ± 0.0	10 ± 0.0
*Klebsiella pneumoniae*	10 ± 0.0	11 ± 0.0	9 ± 0.0	9 ± 0.0	8 ± 0.0	9 ± 0.0	9 ± 0.0	9 ± 0.0
*Enterococcus faecalis*	11 ± 0.0	11 ± 0.0	10 ± 0.0	9 ± 0.0	9 ± 0.0	10 ± 0.0	12 ± 0.0	12 ± 0.0
*Escherichia coli*	9 ± 0.5	10 ± 0.5	8 ± 0.0	10 ± 0.0	9 ± 0.0	9 ± 0.0	11 ± 0.5	11 ± 0.5
*Salmonella enteritidis*	9 ± 0.0	11 ± 0.0	9 ± 0.0	10 ± 0.0	10 ± 0.0	11 ± 0.0	9 ± 0.0	9 ± 0.0
*Proteus vulgaris*	9 ± 0.0	9 ± 0.0	8 ± 0.0	8 ± 0.0	8 ± 0.0	8 ± 0.0	10 ± 0.0	10 ± 0.0
*Pseudomonas aeruginosa*	11 ± 0.0	11 ± 0.0	11 ± 0.0	10 ± 0.0	10 ± 0.0	10 ± 0.0	12 ± 0.0	14 ± 0.0
*Candida albicans*	10 ± 0.5	11 ± 0.5	10 ± 0.5	10 ± 0.5	8 ± 0.5	9 ± 0.5	10 ± 0.0	11 ± 0.0
*Bacillus* *subtilis*	9 ± 0.0	9 ± 0.0	9 ± 0.0	10 ± 0.0	9 ± 0.0	9 ± 0.0	9 ± 0.0	10 ± 0.0
*Saccharomyces cerevisiae*	15 ± 0.0	16 ± 0.0	14 ± 0.5	14 ± 0.5	13 ± 0.0	13 ± 0.0	18 ± 0.0	18 ± 0.0
*Aspergillus niger*	20 ± 0.0	22 ± 0.0	20 ± 0.0	20 ± 0.0	20 ± 0.0	20 ± 0.0	20 ± 0.0	20 ± 0.0
*Penicillium chrysogenum*	18 ± 0.0	19 ± 0.0	21 ± 0.0	20 ± 0.0	18 ± 0.0	19 ± 0.0	20 ± 0.0	20 ± 0.0
*Mucor* spp.	15 ± 0.0	13 ± 0.0	12 ± 0.0	13 ± 0.0	14 ± 0.0	14 ± 0.0	12 ± 0.0	13 ± 0.0
*Rhizopus* spp.	16 ± 0.0	14 ± 0.0	14 ± 0.0	12 ± 0.0	16 ± 0.0	14 ± 0.0	13 ± 0.0	18 ± 0.0

## Data Availability

Data are contained within the article.

## References

[1] Zandi P., Basu S.K., Cetzal-Ix W., Kordrostami M., Khademi Chalaras S., Bazrkar Khatibai L., El-Shemy H.A. (2017). Fenugreek (*Trigonella foenum-graecum* L.): An Important Medicinal and Aromatic Crop. Active Ingredients from Aromatic and Medicinal Plants.

[2] Basu A., Basu S.K., Kumar A., Sharma M., Chalghoumi R., Hedi A., Solorio-Sánchez F., Balogun M.O., Hafez E.E., Cetzal-Ix W. (2014). Fenugreek (*Trigonella foenum-graecum* L.), A Potential New Crop for Latin America. Am. Open Access J. Soc. Issues Humanit..

[3] GBIF.org GBIF Backbone Taxonomy: *Trigonella foenum-graecum* L. https://www.gbif.org/taxon/58R6D.

[4] Editorial Characteristics of Fenugreek Plant. https://www.botanical-online.com/en/botany/fenugreek-plant.

[5] Kaplan M., Kaymaz E., Varol I.S., Ciftci B., Gokalp Z. (2024). Herbage Yield and Biochemical Characterization of Fenugreek (*Trigonella foenum-graecum* L.) Under Different Irrigation Levels. Ind. Crops Prod..

[6] Zandi P., Basu S.K., Khatibani L.B., Balogun M.O., Aremu M.O., Sharma M., Kumar A., Sengupta R., Li X., Li Y. (2014). Fenugreek (*Trigonella foenum-graecum* L.) Seed: A Review of Physiological and Biochemical Properties and Their Genetic Improvement. Acta Physiol. Plant..

[7] Acharya S.N., Thomas J.E., Basu S.K. (2008). Fenugreek, an Alternative Crop for Semiarid Regions of North America. Crop. Sci..

[8] Baset M., Ali T., Elshamy H., El Sadek A., Sami D., Badawy M., Abou-Zekry S., Heiba H., Saadeldin M., Abdellatif A. (2020). Anti-diabetic effects of fenugreek (*Trigonella foenum-graecum*): A comparison between oral and intraperitoneal administration—An animal study. Int. J. Funct. Nutr..

[9] Hamza N., Berke B., Cheze C., Le Garrec R., Umar A., Agli A.-N., Lassalle R., Jové J., Gin H., Moore N. (2012). Preventive and curative effect of *Trigonella foenum-graecum* L. seeds in C57BL/6J models of type 2 diabetes induced by high-fat diet. J. Ethnopharm..

[10] Bahmani M., Shirzad H., Mirhosseini M., Mesripour A., Rafieian-Kopaei M. (2015). A Review on Ethnobotanical and Therapeutic Uses of Fenugreek (*Trigonella foenum-graceum L*). J. Evid.-Based Complement. Altern. Med..

[11] Varshney H., Siddique Y.H. (2023). Medicinal Properties of Fenugreek: A Review. Open Biol. J..

[12] Faisal Z., Irfan R., Akram N., Manzoor H.M.I., Aabdi M.A., Anwar M.J., Khawar S., Saif A., Shah Y.A., Afzaal M. (2024). The Multifaceted Potential of Fenugreek Seeds: From Health Benefits to Food and Nanotechnology Applications. Food Sci. Nutr..

[13] Suja Pandian R., Anuradha C.V., Viswanathan P. (2002). Gastroprotective Effect of Fenugreek Seeds (*Trigonella foenum graecum*) on Experimental Gastric Ulcer in Rats. J. Ethnopharm..

[14] Jeevanandam J., Barhoum A., Chan Y.S., Dufresne A., Danquah M.K. (2018). Review on Nanoparticles and Nanostructured Materials: History, Sources, Toxicity and Regulations. Beilstein J. Nanotechnol..

[15] Virmani I., Sasi C., Priyadarshini E., Kumar R., Sharma S.K., Singh G.P., Pachwarya R.B., Paulraj R., Barabadi H., Saravanan M. (2019). Comparative Anticancer Potential of Biologically and Chemically Synthesized Gold Nanoparticles. J. Clust. Sci..

[16] Jain A.S., Pawar P.S., Sarkar A., Junnuthula V., Dyawanapelly S. (2021). Bionanofactories for Green Synthesis of Silver Nanoparticles: Toward Antimicrobial Applications. Int. J. Mol. Sci..

[17] Ivanova A., Todorova M., Petrov D., Gledacheva V., Stefanova I., Milusheva M., Slavchev V., Kostadinova G., Petkova Z., Teneva O. (2026). Biogenic Synthesis, Structural Characterization, and Biological Evaluation of Nanoparticles Derived from *Chlorella vulgaris* Ethanolic Extract. Nanomaterials.

[18] Ivanova A., Todorova M., Petrov D., Petkova Z., Teneva O., Antova G., Angelova-Romova M., Yanakieva V., Tsoneva S., Gledacheva V. (2025). From Spirulina Platensis to Nanomaterials: A Comparative Study of AgNPs Obtained from Two Extracts. Nanomaterials.

[19] Ahmed M., Marrez D.A., Abdelmoeen N.M., Mahmoud E.A., Abdel-Shakur Ali M., Decsi K., Tóth Z. (2023). Proximate Analysis of Moringa oleifera Leaves and the Antimicrobial Activities of Successive Leaf Ethanolic and Aqueous Extracts Compared with Green Chemically Synthesized Ag-NPs and Crude Aqueous Extract Against Some Pathogens. Int. J. Mol. Sci..

[20] Panova N., Gerasimova A., Tumbarski Y., Ivanov I., Todorova M., Dincheva I., Gentscheva G., Gledacheva V., Slavchev V., Stefanova I. (2025). Metabolic Profile, Antioxidant, Antimicrobial, Contractile, and Anti-Inflammatory Potential of Moringa oleifera Leaves (India). Life.

[21] Alshehri A.A., Malik M.A. (2020). Phytomediated Photo-Induced Green Synthesis of Silver Nanoparticles Using *Matricaria chamomilla* L. and Its Catalytic Activity Against Rhodamine B. Biomolecules.

[22] Macovei I., Luca S.V., Skalicka-Woźniak K., Sacarescu L., Pascariu P., Ghilan A., Doroftei F., Ursu E.-L., Rimbu C.M., Horhogea C.E. (2022). Phyto-Functionalized Silver Nanoparticles Derived from Conifer Bark Extracts and Evaluation of Their Antimicrobial and Cytogenotoxic Effects. Molecules.

[23] Corciovă A., Mircea C., Burlec A.F., Fifere A., Moleavin I.T., Sarghi A., Tuchiluș C., Ivănescu B., Macovei I. (2022). Green Synthesis and Characterization of Silver Nanoparticles Using a *Lythrum salicaria* Extract and In Vitro Exploration of Their Biological Activities. Life.

[24] Jespersen B., Tykocki N.R., Watts S.W., Cobbett P.J. (2015). Measurement of Smooth Muscle Function in the Isolated Tissue Bath-Applications to Pharmacology Research. J. Vis. Exp..

[25] Milusheva M., Todorova M., Gledacheva V., Stefanova I., Feizi-Dehnayebi M., Pencheva M., Nedialkov P., Tumbarski Y., Yanakieva V., Tsoneva S. (2023). Novel Anthranilic Acid Hybrids—An Alternative Weapon against Inflammatory Diseases. Pharmaceuticals.

[26] Yanakieva V., Parzhanova A., Dimitrov D., Vasileva I., Raeva P., Ivanova S. (2023). Study on the Antimicrobial Activity of Medicinal Plant Extracts and Emulsion Products with Integrated Herbal Extracts. Agric. For..

[27] Goyal S., Gupta N., Chatterjee S. (2016). Investigating Therapeutic Potential of *Trigonella foenum-graecum* L. as Our Defense Mechanism Against Several Human Diseases. J. Toxicol..

[28] Younesy S., Amiraliakbari S., Esmaeili S., Alavimajd H., Nouraei S. (2025). Effects of Fenugreek Seed on the Severity and Systemic Symptoms of Dysmenorrhea. J. Reprod. Infertil..

[29] Lininger S.W., Wright J.V. (1998). The Natural Pharmacy.

[30] Fetrow C.W. (2004). Professional’s Handbook of Complementary & Alternative Medicines.

[31] Srichamroen A., Thomson R., Field C.J., Basu T.K. (2009). In Vitro Intestinal Glucose Uptake Is Inhibited by Galactomannan from Canadian Fenugreek Seed (*Trigonella foenum graecum* L) in Genetically Lean and Obese Rats. Nutr. Res..

[32] Kaviarasan S., Naik G.H., Gangabhagirathi R., Anuradha C.V., Priyadarsini K.I. (2007). In Vitro Studies on Antiradical and Antioxidant Activities of Fenugreek (*Trigonella foenum graecum*) Seeds. Food Chem..

[33] Hedlund P., Aszódi A., Pfeifer A., Alm P., Hofmann F., Ahmad M., Reinhard Fässler R., Andersson K.-E. (2000). Erectile Dysfunction in Cyclic GMP-Dependent Kinase I-Deficient Mice. Proc. Nat. Acad. Sci. USA.

[34] Ghayur M.N., Abdalla M., Khalid A., Ahmad S., Gilani A.H. (2022). *Trigonella foenum-graecum* Methanolic Extract on Isolated Smooth Muscles and Acetylcholinesterase Enzyme: An In Vitro and Mechanistic in Silico Investigation. BioMed Res. Int..

[35] Khan M., Shaik M.R., Adil S.F., Khan S.T., Al-Warthan A., Siddiqui M.R.H., Tahir M.N., Tremel W. (2018). Plant Extracts as Green Reductants for the Synthesis of Silver Nanoparticles: Lessons from Chemical Synthesis. Dalton Trans..

[36] Rodríguez De Luna S.L., Ramírez-Garza R.E., Serna Saldívar S.O. (2020). Environmentally Friendly Methods for Flavonoid Extraction from Plant Material: Impact of Their Operating Conditions on Yield and Antioxidant Properties. Sci. World J..

[37] Yadi M., Mostafavi E., Saleh B., Davaran S., Aliyeva I., Khalilov R., Nikzamir M., Nikzamir N., Akbarzadeh A., Panahi Y. (2018). Current Developments in Green Synthesis of Metallic Nanoparticles Using Plant Extracts: A Review. Artif. Cells Nanomed. Biotechnol..

[38] Kalantari K., Mostafavi E., Afifi A.M., Izadiyan Z., Jahangirian H., Rafiee-Moghaddam R., Webster T.J. (2020). Wound Dressings Functionalized with Silver Nanoparticles: Promises and Pitfalls. Nanoscale.

[39] Lotfollahzadeh R., Yari M., Sedaghat S., Delbari A.S. (2021). Biosynthesis and Characterization of Silver Nanoparticles for the Removal of Amoxicillin from Aqueous Solutions Using Oenothera Biennis Water Extract. J. Nanostructure Chem..

[40] El-Bahy G.M.S. (2005). FTIR and Raman Spectroscopic Study of Fenugreek (*Trigonella foenum graecum* L.) Seeds. J. Appl. Spectrosc..

[41] Vanlalveni C., Lallianrawna S., Biswas A., Selvaraj M., Changmai B., Rokhum S.L. (2022). Green Synthesis of Silver Nanoparticles Using Plant Extracts and Their Antimicrobial Activities: A Review of Recent Literature. RSC Adv..

[42] Wirwis A., Sadowski Z. (2023). Green Synthesis of Silver Nanoparticles: Optimizing Green Tea Leaf Extraction for Enhanced Physicochemical Properties. ACS Omega.

[43] Sahu N., Soni D., Chandrashekhar B., Satpute D.B., Saravanadevi S., Sarangi B.K., Pandey R.A. (2016). Synthesis of Silver Nanoparticles Using Flavonoids: Hesperidin, Naringin and Diosmin, and Their Antibacterial Effects and Cytotoxicity. Int. Nano Lett..

[44] Liu L., Yu C., Ahmad S., Ri C., Tang J. (2023). Preferential Role of Distinct Phytochemicals in Biosynthesis and Antibacterial Activity of Silver Nanoparticles. J. Environ. Manag..

[45] Meena R.K., Chouhan N. (2015). Biosynthesis of silver nanoparticles from plant (fenugreek seeds) reducing method and their optical properties. Res. J. Recent Sci..

[46] Ahani M., Khatibzadeh M. (2021). Green Synthesis of Silver Nanoparticles Using Gallic Acid as Reducing and Capping Agent: Effect of pH and Gallic Acid Concentration on Average Particle Size and Stability. Inorg. Nano-Met. Chem..

[47] Awad M.A., Virk P., Hendi A.A., Ortashi K.M., AlMasoud N., Alomar T.S. (2023). Role of Biosynthesized Silver Nanoparticles with *Trigonella foenum-graecum* Seeds in Wastewater Treatment. Processes.

[48] Pochapski D.J., Carvalho dos Santos C., Leite G.W., Pulcinelli S.H., Santilli C.V. (2021). Zeta Potential and Colloidal Stability Predictions for Inorganic Nanoparticle Dispersions: Effects of Experimental Conditions and Electrokinetic Models on the Interpretation of Results. Langmuir.

[49] Ojha I., Saud P.S., Jaishi D.R., Rosyara Y.R., Ojha A., Devi N.R., Joshi P.R., Pant H.R. (2026). Plant-Mediated Synthesis of Silver Nanoparticles Using *Alcea rosea* Leaf Aqueous Extract and Evaluation of the Biological Activities. Sci. Rep..

[50] Shahabadi N., Shalmashi K., Shokraei S., Soltani L. (2026). Plant-Mediated Fabrication of Silver Nanoparticles Using *Elaeagnus angustifolia* Flowers: In Vitro Evaluation of Antibacterial, Antifungal, Antioxidant, and Toxic Effects and Biomolecular Interactions With Ct-DNA and HSA. Bioinorg. Chem. Appl..

[51] Ravichandran V., Vasanthi S., Shalini S., Shah S.A.A., Tripathy M., Paliwal N. (2019). Green Synthesis, Characterization, Antibacterial, Antioxidant and Photocatalytic Activity of *Parkia speciosa* Leaves Extract Mediated Silver Nanoparticles. Results Phys..

[52] Moond M., Singh S., Sangwan S., Rani S., Beniwal A., Rani J., Kumari A., Rani I., Devi P. (2023). Phytofabrication of Silver Nanoparticles Using *Trigonella foenum-graceum* L. Leaf and Evaluation of Its Antimicrobial and Antioxidant Activities. Int. J. Mol. Sci..

[53] Chomentowski A., Drygalski K., Kleszczewski T., Berczyńska M., Tylicka M., Kapała J., Raciborska A., Zubrzycki P., Hady H.R., Modzelewska B. (2025). Polyphenols as Modulators of Gastrointestinal Motility: Mechanistic Insights from Multi-Model Studies. Pharmaceuticals.

[54] Albaker W.I. (2023). Fenugreek and Its Effects on Muscle Performance: A Systematic Review. J. Pers. Med..

[55] Sharma V.K., Yngard R.A., Lin Y. (2009). Silver Nanoparticles: Green Synthesis and Their Antimicrobial Activities. Adv. Colloid Interface Sci..

[56] Habschied K., Krstanović V., Zdunić Z., Babić J., Mastanjević K., Šarić G.K. (2021). Mycotoxins Biocontrol Methods for Healthier Crops and Stored Products. J. Fungi.

[57] Haque M.A., Wang Y., Shen Z., Li X., Saleemi M.K., He C. (2020). Mycotoxin Contamination and Control Strategy in Human, Domestic Animal and Poultry: A Review. Microb. Pathog..

[58] Ramírez-Camejo L.A., Zuluaga-Montero A., Morris V., Rodríguez J.A., Lázaro-Escudero M.T., Bayman P. (2022). Fungal Diversity in Sahara Dust: *Aspergillus sydowii* and Other Opportunistic Pathogens. Aerobiologia.

[59] Salazar F., Bignell E., Brown G.D., Cook P.C., Warris A. (2021). Pathogenesis of Respiratory Viral and Fungal Coinfections. Clin. Microbiol. Rev..

[60] Kumar P., Kausar M.A., Singh A.B., Singh R. (2021). Biological Contaminants in the Indoor Air Environment and Their Impacts on Human Health. Air Qual. Atmos. Health.

[61] Dacrory S., Hashem A.H., Hasanin M. (2021). Synthesis of Cellulose Based Amino Acid Functionalized Nano-Biocomplex: Characterization, Antifungal Activity, Molecular Docking and Hemocompatibility. Environ. Nanotechnol. Monit. Manag..

[62] Miller A.S., Wilmott R.W. (2019). The Pulmonary Mycoses.

[63] Hendrickson J.A., Hu C., Aitken S.L., Beyda N. (2019). Antifungal Resistance: A Concerning Trend for the Present and Future. Curr. Infect. Dis. Rep..

[64] Visagie C.M., Houbraken J., Frisvad J.C., Hong S.-B., Klaassen C.H.W., Perrone G., Seifert K.A., Varga J., Yaguchi T., Samson R.A. (2014). Identification and Nomenclature of the Genus Penicillium. Stud. Mycol..

[65] Guzmán-Chávez F., Zwahlen R.D., Bovenberg R.A.L., Driessen A.J.M. (2018). Engineering of the Filamentous Fungus *Penicillium chrysogenum* as Cell Factory for Natural Products. Front. Microbiol..

[66] D’Antonio D., Violante B., Farina C., Sacco R., Angelucci D., Masciulli M., Iacone A., Romano F. (1998). Necrotizing Pneumonia Caused by *Penicillium chrysogenum*. J. Clin. Microbiol..

[67] Rios G., Castillo S.B.J., Soto A., De Ajanel M.E.C., Aguilar J., Castellanos J.L.A., Cifuentes J., Conde-Pereira C. (2021). Acute Respiratory Failure with Fatal Outcome due to *Penicillium chrysogenum* in a Man from Guatemala with a Seminoma (Germ Cell Tumor). Chest.

[68] Hashem A.H., Saied E., Amin B.H., Alotibi F.O., Al-Askar A.A., Arishi A.A., Elkady F.M., Elbahnasawy M.A. (2022). Antifungal Activity of Biosynthesized Silver Nanoparticles (AgNPs) Against *Aspergilli* Causing Aspergillosis: Ultrastructure Study. J. Funct. Biomater..

[69] Ghoshal G., Singh M. (2022). Characterization of silver nano-particles synthesized using fenugreek leave extract and its antibacterial activity. Mater. Sci. Energy Technol..

[70] Slavin Y.N., Bach H. (2022). Mechanisms of Antifungal Properties of Metal Nanoparticles. Nanomaterials.

[71] Westmeier D., Solouk-Saran D., Vallet C., Siemer S., Docter D., Götz H., Männ L., Hasenberg A., Hahlbrock A., Erler K. (2018). Nanoparticle Decoration Impacts Airborne Fungal Pathobiology. Proc. Natl. Acad. Sci. USA.

